# Oncogenic KRAS signaling drives evasion of innate immune surveillance in lung adenocarcinoma by activating CD47

**DOI:** 10.1172/JCI153470

**Published:** 2023-01-17

**Authors:** Huanhuan Hu, Rongjie Cheng, Yanbo Wang, Xiaojun Wang, Jianzhuang Wu, Yan Kong, Shoubin Zhan, Zhen Zhou, Hongyu Zhu, Ranran Yu, Gaoli Liang, Qingyan Wang, Xiaoju Zhu, Chen-Yu Zhang, Rong Yin, Chao Yan, Xi Chen

**Affiliations:** 1 State Key Laboratory of Pharmaceutical Biotechnology, Jiangsu Engineering Research Center for MicroRNA Biology and Biotechnology, NJU Advanced Institute of Life Sciences (NAILS), School of Life Sciences, and; 2 Chemistry and Biomedicine Innovation Center (ChemBIC), Nanjing University, Nanjing, China.; 3 Cancer Stem Cell Laboratory, The Breast Cancer Now Toby Robins Research Centre, The Institute of Cancer Research, London, United Kingdom.; 4 Department of Thoracic Surgery, Jiangsu Key Laboratory of Molecular and Translational Cancer Research, Jiangsu Cancer Hospital and Nanjing Medical University Affiliated Cancer Hospital and Jiangsu Institute of Cancer Research, Nanjing, China.; 5 Research Unit of Extracellular RNA, Chinese Academy of Medical Sciences, Nanjing, Jiangsu, China.; 6 Institute of Artificial Intelligence Biomedicine, Nanjing University, Nanjing, China.; 7 Biobank of Lung Cancer, Jiangsu Biobank of Clinical Resources, Nanjing, China.; 8 Collaborative Innovation Centre for Cancer Personalized Medicine, Nanjing Medical University, Nanjing, China.; 9 Pingshan Translational Medicine Center, Shenzhen Bay Laboratory, Shenzhen, Guangdong, China.

**Keywords:** Oncology, Pulmonology, Cancer immunotherapy, Lung cancer, Oncogenes

## Abstract

*KRAS* is one of the most frequently activated oncogenes in human cancers. Although the role of *KRAS* mutation in tumorigenesis and tumor maintenance has been extensively studied, the relationship between KRAS and the tumor immune microenvironment is not fully understood. Here, we identified a role of KRAS in driving tumor evasion from innate immune surveillance. In samples of lung adenocarcinoma from patients and *Kras*-driven genetic mouse models of lung cancer, mutant KRAS activated the expression of cluster of differentiation 47 (CD47), an antiphagocytic signal in cancer cells, leading to decreased phagocytosis of cancer cells by macrophages. Mechanistically, mutant KRAS activated PI3K/STAT3 signaling, which restrained miR-34a expression and relieved the posttranscriptional repression of miR-34a on CD47. In 3 independent cohorts of patients with lung cancer, the *KRAS* mutation status positively correlated with CD47 expression. Therapeutically, disruption of the KRAS/CD47 signaling axis with *KRAS* siRNA, the KRAS^G12C^ inhibitor AMG 510, or a miR-34a mimic suppressed CD47 expression, enhanced the phagocytic capacity of macrophages, and restored innate immune surveillance. Our results reveal a direct mechanistic link between active KRAS and innate immune evasion and identify CD47 as a major effector underlying the KRAS-mediated immunosuppressive tumor microenvironment.

## Graphical abstract

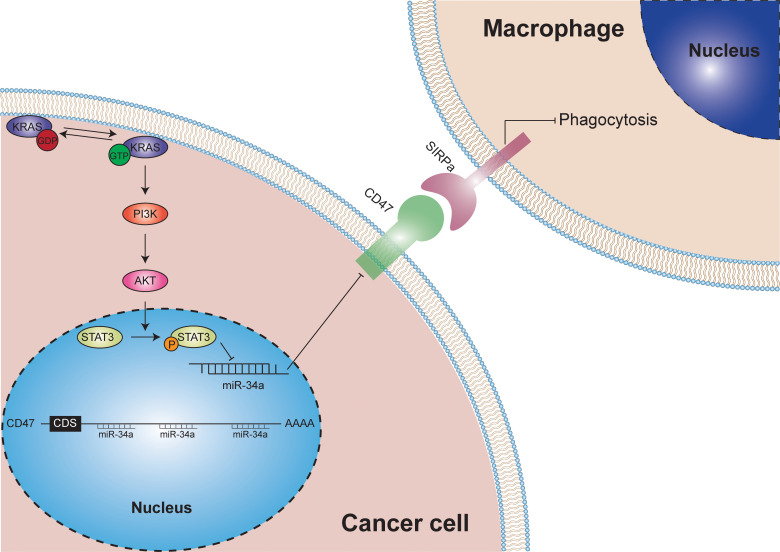

## Introduction

Mutations of the *KRAS* gene are the most common drivers of tumor development across a spectrum of human cancers, such as cancers of the lung, colon, and pancreas (1–3). For example, approximately 30% of lung adenocarcinomas harbor *KRAS* mutations, with *KRAS^G12C^* being the most common subtype (12%–15%) (4). At the molecular level, KRAS proteins with activating mutations abrogate the GTPase activity and are locked in the GTP-bound hyperactive state, leading to constitutive activation of downstream proproliferative and prosurvival pathways such as RAF/MEK/ERK and PI3K/AKT (5, 6). Despite 40 years of intensive efforts to develop targeted therapy for the KRAS oncoprotein, only AMG 510, a covalent inhibitor of the KRAS^G12C^ mutation, has just been approved by the FDA in May 2021; and targeting other KRAS mutations is still considered “mission impossible” (7, 8). Understanding how KRAS mutations drive cancer pathogenesis and developing new interventional strategies are the major priorities for conquering *KRAS*-driven cancers.

An emerging and exciting new direction may come from recent advances in our understanding of the relationship between KRAS mutations and tumor immune evasion. Tumor cells often overexpress immune checkpoint molecules to escape immune surveillance. As 2 representative checkpoint molecules often overexpressed on the surface of cancer cells, programmed death ligand 1 (PD-L1) signals to T cells to evade attacks from the adaptive immune system, whereas cluster of differentiation 47 (CD47) is a critical antiphagocytosis signal to macrophages in the innate immune system (9–11). Recently, Coelho et al. unveiled a novel function of oncogenic KRAS signaling (mainly the KRAS^G12V^ mutation) in driving tumor cell PD-L1 expression, thereby impairing adaptive immune surveillance and favoring tumor growth (12). Canon et al. also showed that the KRAS^G12C^ inhibitor AMG 510 could drive antitumor immunity through inhibition of PD-L1 signaling (13). While KRAS-mediated evasion of the adaptive immune response has been increasingly recognized, whether KRAS plays a role in innate immune surveillance has not been delineated.

The innate immune system plays an important role in tumor surveillance mainly through the phagocytic activity of macrophages. In the early stage of tumor formation, macrophages actively infiltrate tumor tissues and phagocytose tumor cells; later on, their phagocytic capacity is gradually suppressed by tumor-derived inhibitory signals (14). As the most studied antiphagocytosis signal in the tumor microenvironment, CD47 has been shown to be overexpressed on the surface of many types of cancer cells. Binding of CD47 to its receptor signal regulatory protein α (SIRPα) on macrophages inhibits macrophage-mediated phagocytosis (15). High CD47 expression in the tumor microenvironment has also been associated with poor patient prognosis in various cancer types (16–19). Moreover, therapeutic blockade of the CD47/SIRPα axis using anti-CD47 mAbs has demonstrated efficacy in a variety of preclinical models and is currently in clinical trials for both leukemia and solid tumors (20). However, little is known about the genetic and epigenetic regulation of the expression of CD47 in cancer cells.

In this study, we showed that oncogenic KRAS mutations interact with the innate immune system in the context of lung adenocarcinoma progression by promoting tumor cell evasion from macrophage phagocytosis. We also dissected the underlying molecular mechanisms and revealed that KRAS mutations could directly activate CD47 in cancer cells to inhibit the activity of macrophages, thereby leading to innate immune evasion and aggressive tumor progression. We therefore propose that *KRAS* mutation status could serve as a biomarker for anti-CD47 cancer immunotherapy.

## Results

### KRAS mutations render lung cancer cells insensitive to phagocytosis by macrophages.

**Figure 1:**
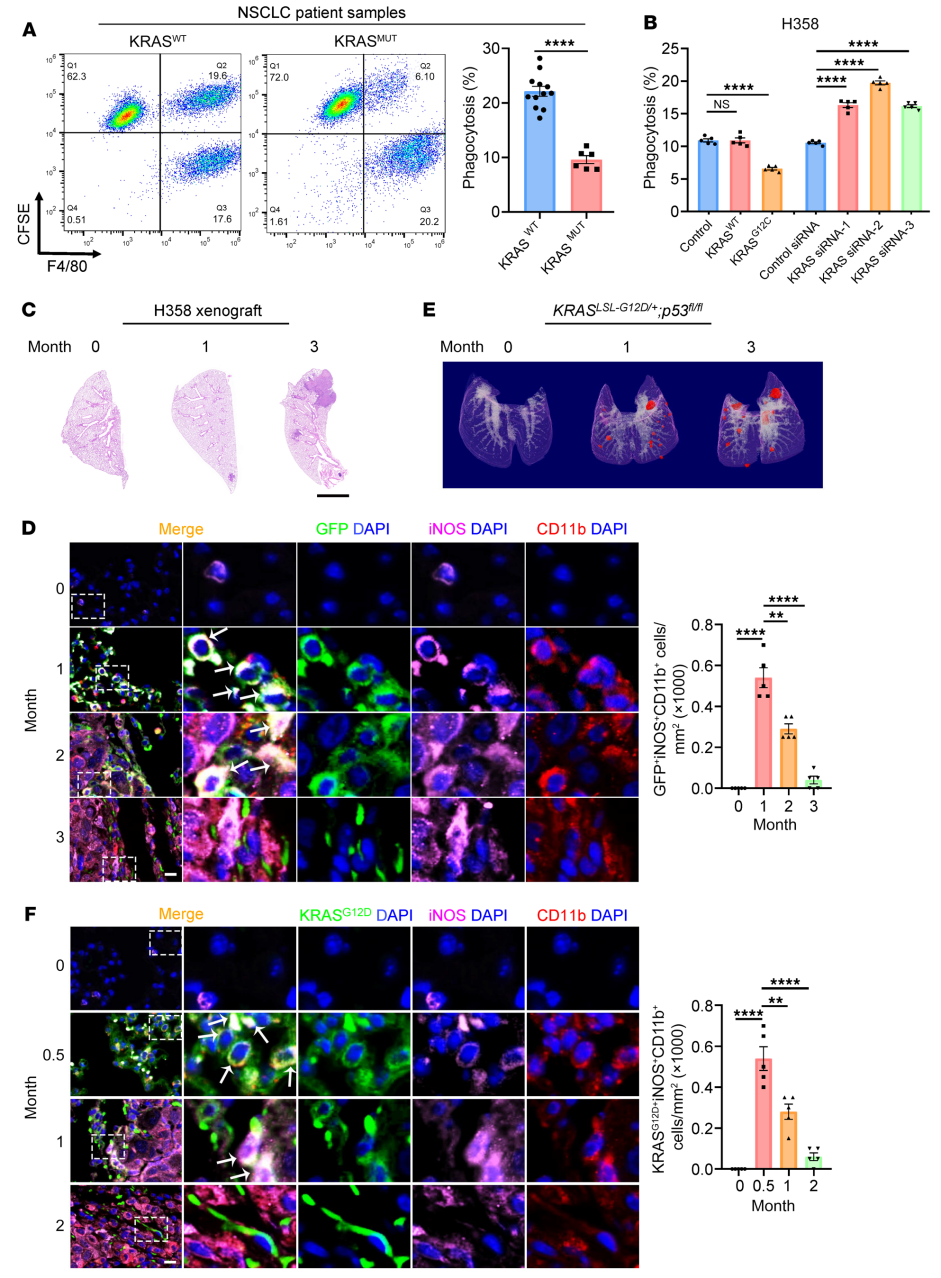
Oncogenic *KRAS* mutations render lung cancer cells insensitive to macrophage phagocytosis. (**A**) Tumor cells were isolated from 12 *KRAS^WT^* and 6 *KRAS^MUT^* lung adenocarcinoma patients, labeled with the fluorescent dye CFSE, incubated with human peripheral blood monocyte–derived macrophages for 2 hours, stained with F4/80, and analyzed by flow cytometry. Phagocytosis rate was calculated as the percentage of CFSE^+^F4/80^+^ cells among CFSE^+^ cells. Representative FACS results and quantification for all patients are shown. (**B**) *KRAS^G12C^* H358 cells were transfected with plasmids expressing KRAS^WT^ or KRAS^G12C^ or with 3 *KRAS* siRNAs. After 48 hours, the cells were subjected to a phagocytosis assay similar to that in **A**. Quantitative analysis (*n* = 5) is shown. NSCLC, non–small cell lung cancer. (**C** and **D**) Macrophage phagocytosis of H358 cells gradually decreased with tumor progression in vivo. An animal model of lung cancer was established by tail-vein injection of EGFP-labeled H358 cells into nude mice. (**C**) Representative images of H&E-stained lung sections at different time points are shown. Scale bar: 2 mm. (**D**) Macrophage infiltration into lung tumor tissue was assessed by CD11b (red) and iNOS (purple) staining. Representative images and quantification results (*n* = 5) are shown. Arrows indicate GFP^+^iNOS^+^CD11b^+^ cells. Scale bar: 50 μm. Original magnification, ×40 (enlarged insets). (**E** and **F**) Macrophage phagocytosis of tumor cells gradually decreased with tumor progression in genetic models of lung cancer. *Kras^LSL-G12D/+^ p53^fl/fl^* mice were intratracheally administered adeno-Cre to trigger pulmonary adenocarcinoma formation. Tumor growth was monitored by micro-CT at different time points. (**E**) Representative 3D reconstructions of mouse lungs. Tumors are shown in red. (**F**) Macrophage infiltration was assessed by staining for CD11b (red), iNOS (purple), and KRAS^G12D^ (green). Representative images and quantification results (*n* = 5) are shown. Arrows indicate KRAS^G12D+^iNOS^+^CD11b^+^ cells. Scale bar: 50 μm. Original magnification, ×40 (enlarged insets). Data are shown as the mean ± SEM. **P < 0.01 and ****P < 0.0001, by unpaired *t* test (**A**) or 1-way ANOVA (**B**, **D**, and **F**).

We set out to investigate whether *KRAS* mutations could lead to an impaired innate immune response against tumors in 18 patients with lung adenocarcinoma (12 harbored WT *KRAS* [*KRAS^WT^*] and 6 had *KRAS* mutations). Primary tumor cells freshly isolated from surgically removed tumor tissues were fluorescently labeled with CFSE and cocultured with human peripheral blood monocyte–derived macrophages and analyzed by FACS to quantify cancer cells that were phagocytosed by macrophages. Tumors cells derived from patients with the *KRAS* mutation showed significantly less phagocytosis by macrophages (Figure 1A and Supplemental Figure 1A; supplemental material available online with this article; https://doi.org/10.1172/JCI153470DS1). We carried out a similar phagocytosis assay using the human lung cancer cell line H358 harboring the *KRAS^G12C^* mutation. We cocultured CFSE-labeled H358 cells with human peripheral blood monocyte–derived macrophages and analyzed them by FACS or fluorescence microscopy. We found that overexpression of KRAS^G12C^, but not KRAS^WT^, led to decreased phagocytosis of H358 cells by macrophages, whereas siRNA knockdown of *KRAS* increased phagocytosis (Figure 1B and Supplemental Figure 1, B–F). We also established a lung colonization model by tail-vein injection of EGFP-labeled H358 cells into nude mice (Figure 1C). We analyzed the infiltration of macrophages in mouse lung tumors by immunofluorescence staining with antibodies against the typical myeloid marker CD11b and the tumoricidal M1 macrophage marker inducible NOS (iNOS). Although the M1 macrophage population (CD11b^+^iNOS^+^ or iNOS^+^TNF-α^+^ by IHC) was continuously present in the tumor tissue over the 3-month experiment period (Figure 1D and Supplemental Figure 2A), the colocalization of the CD11b/iNOS signal and the EGFP signal displayed a gradual decrease over time (Figure 1D), indicating impaired macrophage phagocytosis of H358 tumor cells in vivo.

We further confirmed this observation in 2 *Kras*-driven genetically engineered mouse models of lung cancer: the Lox-Stop-Lox-*Kras^G12D^* (*Kras^LSL-G12D/+^*) mouse strain and the *Kras^LSL-G12D/+^* *p53^fl/fl^* mouse strain (21, 22). The *Kras^LSL-G12D/+^* mice developed spontaneous, sporadic pulmonary adenocarcinomas following intratracheal administration of the Cre-expressing adenovirus (adeno-Cre) to remove the Stop element from the *Kras^G12D^* allele, while the *Kras^LSL-G12D/+^* *p53^fl/fl^* mice exhibited accelerated pulmonary adenocarcinoma formation by concomitant deletion of the *Tp53* tumor suppressor gene. As expected, we observed spontaneous formation of small adenocarcinomas in the lungs of the *Kras^LSL-G12D/+^* mice 5 months after adeno-Cre administration (Supplemental Figure 2, B and C), whereas pulmonary adenocarcinomas were present in the *Kras^LSL-G12D/+^* *p53^fl/fl^* mice at 1 month and substantially increased at 3 months (Figure 1E and Supplemental Figure 3, A and B). We then analyzed the status of macrophage infiltration and tumor phagocytosis in the lung tumors from both *Kras^LSL-G12D/+^* and *Kras^LSL-G12D/+^* *p53^fl/fl^* mice. Similar to the H358 model, we observed a gradual decrease in macrophage phagocytosis of tumor cells over time (Figure 1F and Supplemental Figure 2D) despite the continuous presence of M1 macrophages, which was confirmed by IHC with the M1 macrophage markers iNOS and TNF-α (Supplemental Figure 2E and Supplemental Figure 3C). Together, these results suggested that *KRAS* mutations endowed lung cancer cells with an antiphagocytic capacity during tumor progression.

### CD47 is required for a KRAS-driven antiphagocytic effect in lung cancer.

**Figure 2:**
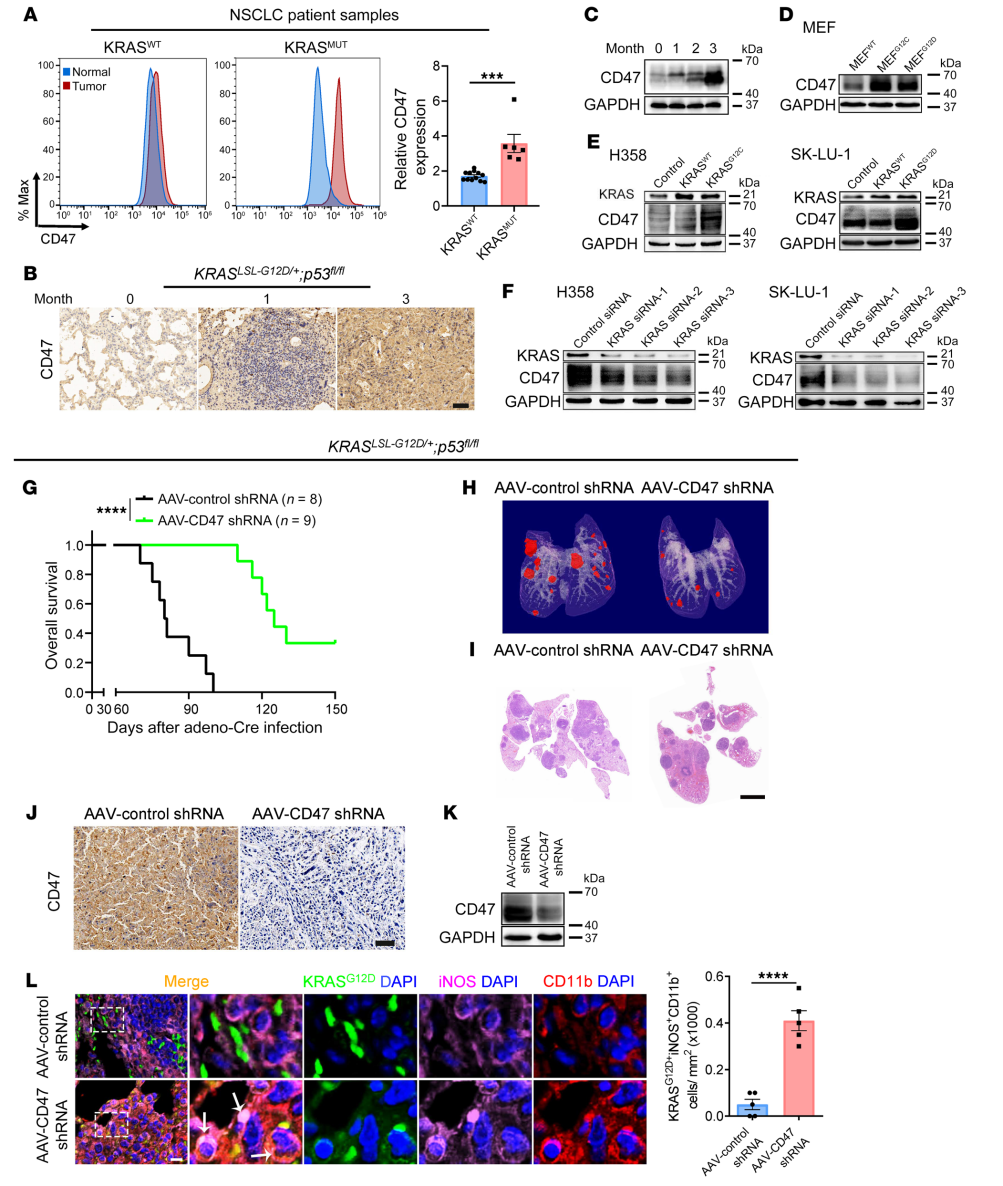
CD47 is required for a *KRAS*-driven antiphagocytic effect in lung cancer. (**A**) Differential expression of CD47 in patients with lung adenocarcinoma with different KRAS mutation statuses. Cancer cells were isolated from 12 patients with *KRAS^WT^* and 6 patients with *KRAS^MUT^* and analyzed for CD47 expression by FACS. Representative FACS results and quantification of all patients are shown. Max, maximum. (**B** and **C**) *Kras^LSL-G12D/+^* *p53^fl/fl^* mice showed a gradual increase in CD47 expression in lung tumor tissues over time. *Kras^LSL-G12D/+^* *p53^fl/fl^* mice were sacrificed at 0, 1, 2, and 3 months, respectively, and analyzed for CD47 expression by IHC (**B**) and immunoblotting (**C**). Scale bar: 20 μm. (**D**) Immunoblot analysis of CD47 expression in *Ras*-less MEFs overexpressing various KRAS mutations (WT, G12C, or G12D). (**E** and **F**) Effect of KRAS manipulation on CD47 expression in H358 and SK-LU-1 cells. Cells were transfected with plasmids expressing KRAS^WT^, KRAS^G12C^, or KRAS^G12D^ or with 3 *KRAS* siRNAs. After 48 hours, CD47 expression was determined by immunoblotting. (**G**–**L**) Effect of *Cd47* knockdown on *Kras*-driven tumorigenesis and macrophage phagocytosis in vivo. *Kras^LSL-G12D/+^ p53^fl/fl^* mice were intratracheally administered adeno-Cre along with an AAV encoding the shRNA of *Cd47* (AAV-*Cd47* shRNA) or a scrambled negative control shRNA (AAV-control shRNA). (**G**) Kaplan-Meier survival analysis. (**H**) Representative micro-CT visualization of tumors 3 months after administration. (**I**) Representative H&E staining of lung sections. Scale bar: 2 mm. (**J**) Representative IHC staining for CD47. Scale bar: 20 μm. (**K**) Representative immunoblot of CD47 expression in lung tumors. (**L**) Immunofluorescence staining for CD11b (red), iNOS (purple), and KRAS^G12D^ (green) in lung tumors showing an increase in macrophage phagocytosis of tumor cells with *Cd47* shRNA. Representative images and quantification results (*n* = 5 mice) are shown. Arrows indicate KRAS^G12D+^iNOS^+^CD11b^+^ cells. Scale bar: 50 μm. Original magnification, ×40 (enlarged insets). Data are shown as the mean ± SEM. ***P < 0.001 and ****P < 0.0001, by unpaired *t* test (**A** and **L**) or log-rank (Mantel-Cox) test (**G**).

Since cell-surface expression of CD47 is a major mechanism used by cancer cells to evade macrophage phagocytosis, we investigated whether the *KRAS* mutation status could affect CD47 expression in lung tumors. We first analyzed CD47 expression on the surface of tumor cells isolated from the 18 paired lung adenocarcinoma patient samples by flow cytometry. Patients with *KRAS* mutations showed a higher level of CD47 expression on the tumor cell surface (Figure 2A and Supplemental Figure 4A). Next, in both the *Kas^LSL-G12D/+^* and *Kras^LSL-G12D/+^* *p53^fl/fl^* mice, CD47 protein expression in the lung also gradually increased over time (Figure 2, B and C, and Supplemental Figure 4, B–E). We then used the *Ras*-less mouse embryonic fibroblast (MEF) model (23) to investigate the role of *Kras* mutations in regulating CD47 expression. We found that CD47 protein levels were significantly elevated in the *Ras*-less MEFs stably overexpressing KRAS^G12C^ or KRAS^G12D^ compared with levels in KRAS^WT^ MEFs (Figure 2D and Supplemental Figure 5A). Moreover, we used 2 human lung cancer cell lines, H358 (*KRAS^G12C^*) and SK-LU-1 (*KRAS^G12D^*), to further illustrate the potential regulation of CD47 by KRAS. Overexpression of the respective KRAS^MUT^, but not KRAS^WT^, led to a significant induction of CD47 expression in H358 and SK-LU-1 cells (Figure 2E and Supplemental Figure 5, B and C). In contrast, siRNA knockdown of *KRAS* significantly decreased CD47 expression in both cell lines (Figure 2F and Supplemental Figure 5, D and E). These data indicated that *KRAS* mutations could drive CD47 expression both in vitro and in vivo.

To investigate whether CD47 is required for *KRAS*-driven tumor evasion from macrophage phagocytosis, we treated the *Kras^LSL-G12D/+^* *p53^fl/fl^* mice with *Cd47* shRNA delivered by adeno-associated virus (AAV) along with intratracheal administration of adeno-Cre. Knockdown of *Cd47* significantly decreased tumor formation in the lungs of mice and prolonged overall survival (Figure 2, G–K, and Supplemental Figure 6, A–D). Importantly, *Cd47* knockdown also substantially increased the infiltration of M1 macrophages in tumor tissues and the phagocytosis of tumor cells by M1 macrophages in vivo (Figure 2L and Supplemental Figure 6E). Taken together, these data suggested that CD47 was involved in *KRAS*-driven tumor progression by restraining the antitumorigenic properties of macrophages.

### miR-34a is a negative regulator of CD47-mediated antiphagocytic activity.

**Figure 3:**
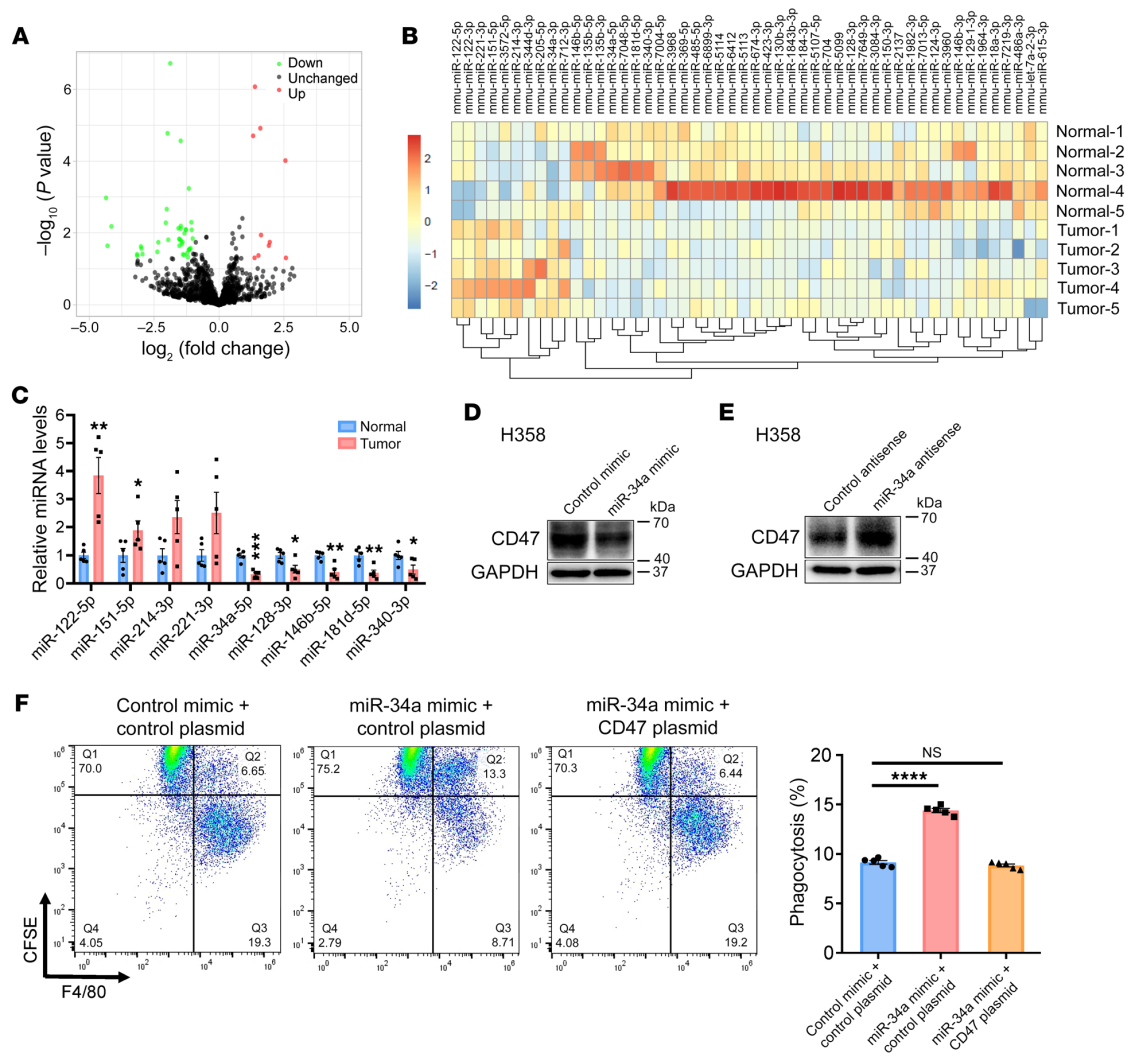
miR-34a restricts CD47 activity and restores the phagocytic function of macrophages in vitro. (**A**) Scatter plot comparison illustrating miRNAs that were differentially expressed between malignant and normal lung tissues from *Kras^LSL-G12D/+^* mice. Down, downregulated; Up, upregulated. (**B**) Dendrogram generated by unsupervised hierarchical cluster analysis showing the separation of tumors from normal tissues based on miRNA profiling (10 upregulated vs. 40 downregulated). (**C**) Quantitative RT-PCR analysis of the 9 most changed miRNAs in **A** (*n* = 5 mice). (**D** and **E**) Effect of the miR-34a mimic or the miR-34a antisense strand on CD47 expression in H358 cells. (**F**) Effect of the miR-34a mimic on macrophage phagocytosis of H358 cells. Cells were transfected with the miR-34a mimic and/or a CD47-expressing plasmid. After 48 hours, cells were subjected to a macrophage phagocytosis assay. Representative FACS images and quantification results (*n* = 5) are shown. Data are shown as the mean ± SEM. *P < 0.05, **P < 0.01, ***P < 0.001, and ****P < 0.0001, by unpaired *t* test (**C**) or 1-way ANOVA (**F**).

Although our data showed a positive correlation between *KRAS* mutation status and CD47 protein levels in both murine and human tumors, *CD47* mRNA levels were unaffected by *KRAS* mutation status (Supplemental Figure 7), suggesting the involvement of posttranscriptional regulatory mechanisms. Since miRNAs represent a fundamental and common posttranscriptional regulator of gene expression (24), we hypothesized that KRAS may regulate CD47 expression through miRNAs. We therefore performed small RNA (sRNA) deep sequencing to determine the alteration of miRNA profiles in lung tumors from *Kras^LSL-G12D/+^* mice compared with normal lung tissues from mice without adeno-Cre treatment. By applying a stringent threshold of a log_2_ fold change of greater than 1 and a significance criterion of *P* of less than 0.05, a total of 10 miRNAs were found to be significantly increased in the lung tumors, whereas 40 miRNAs exhibited a decreasing trend (Figure 3A). Hierarchical clustering also revealed the separation of tumorous from normal tissues based on miRNA profiling (Figure 3B). Subsequently, we confirmed the expression of some top-ranked dysregulated miRNAs (mean reads >500, log_2_ fold change >1 and *P* < 0.01) (Supplemental Table 1) by quantitative reverse transcription PCR (RT-PCR). Seven of the 9 miRNAs were differentially expressed in lung tumors compared with expression in normal tissues (Figure 3C). Because miRNAs usually suppress the expression of their target genes, we focused on the 5 miRNAs that were decreased during tumorigenesis. Using 3 different computational software programs (TargetScan, miRanda, and PicTar), we identified miR-34a-5p (miR-34a), 1 of the 5 most downregulated miRNAs in lung tumors of *Kras^LSL-G12D/+^* mice, as a potential regulator of CD47 expression. We identified a total of 3 specific miR-34a–binding sites in the 3′-UTR) of CD47 (Supplemental Figure 8A).

To further validate the correlation between miR-34a and CD47, we assessed CD47 protein levels in H358 and SK-LU-1 cells after transfection with a miR-34a mimic (synthetic dsRNA oligonucleotide that mimics the precursor of miR-34a) or with miR-34a antisense (single-stranded, chemically modified oligonucleotide designed to specifically bind to and inhibit mature miR-34a) (Supplemental Figure 8, B and C). We found that CD47 protein levels were significantly suppressed by the miR-34a mimic and increased by miR-34a antisense in both H358 and SK-LU-1 cells (Figure 3, D and E, and Supplemental Figure 8, D–G), whereas *CD47* mRNA levels were not affected (Supplemental Figure 8, H and I). Furthermore, direct binding of miR-34a to the 3′-UTR of *CD47* mRNA was also confirmed in a luciferase reporter assay (Supplemental Figure 8J). On the contrary, the other 4 miRNAs that were downregulated in tumor tissues did not affect CD47 expression in the same assay (Supplemental Figure 9). These results demonstrated that miR-34a could directly bind to the 3′-UTR of *CD47* mRNA and inhibit CD47 translation. Next, we evaluated the effect of miR-34a on the antiphagocytic activity of CD47 in lung cancer. Introduction of the miR-34a mimic into H358 cells significantly promoted macrophage-mediated phagocytosis, which was reversed by cotransfection with the CD47 overexpression plasmid (Figure 3F and Supplemental Figure 10, A and B).

**Figure 4:**
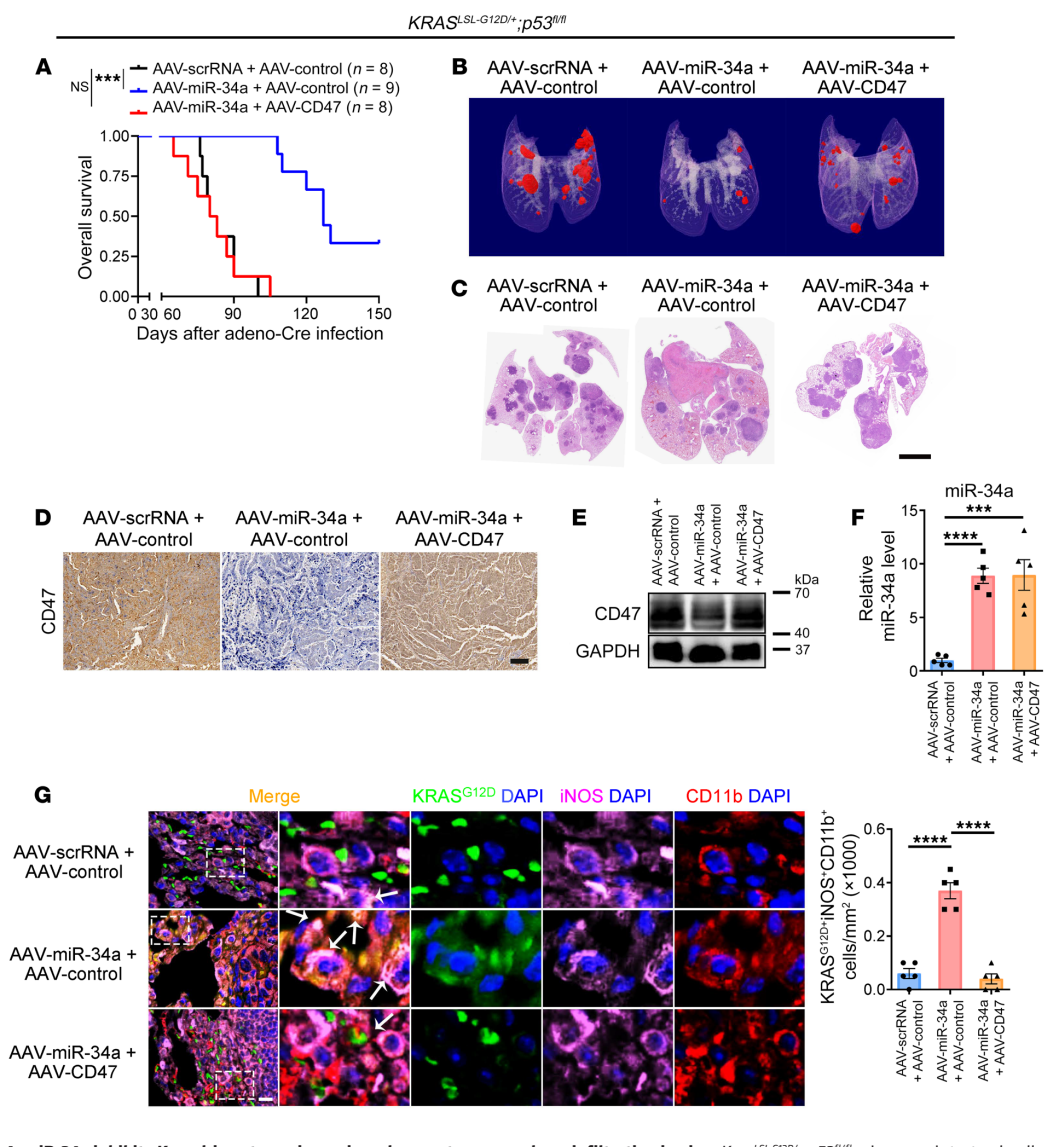
miR-34a inhibits *Kras*-driven tumorigenesis and promotes macrophage infiltration in vivo. *Kras^LSL-G12D/+^p53^fl/fl^* mice were intratracheally administered adeno-Cre along with AAV-miR-34a (or control AAV-scrRNA) and AAV-*CD47* (or AAV-control). (**A**) Kaplan-Meier survival analysis. (**B**) Representative micro-CT visualization of tumors 3 months after administration. (**C**) Representative H&E staining of lung sections. Scale bar: 2 mm. (**D**) Representative IHC staining for CD47 in lung sections. Scale bar: 20 μm. (**E**) Representative immunoblot of CD47 expression in lung tumors. (**F**) miR-34a levels in lung tumors (*n* = 5). (**G**) Immunofluorescence staining for CD11b (red), iNOS (purple), KRAS^G12D^ (green), and DAPI (blue) in lung tumors showing an increase in macrophage phagocytosis of tumor cells with AAV-miR-34a, which was rescued by AAV-CD47. Representative images and quantification results (*n* = 5 mice) are shown. Arrows indicate KRAS^G12D+^iNOS^+^CD11b^+^ cells. Scale bar: 50 μm. Original magnification, ×40 (enlarged insets). Data are shown as the mean ± SEM. ***P < 0.001 and ****P < 0.0001, by 1-way ANOVA (**F** and **G**) or log-rank (Mantel-Cox) test (**A**).

Subsequently, we evaluated the in vivo effects of miR-34a on *KRAS*-driven tumorigenesis and macrophage infiltration. AAV-mediated delivery of miR-34a at the time of intratracheal adeno-Cre administration to *Kras^LSL-G12D/+^* *p53^fl/fl^* mice significantly decreased lung tumor formation and prolonged overall survival, which could be completely rescued by coadministration of the AAV-mediated CD47-overexpressing plasmid (Figure 4, A–C, and Supplemental Figure 10, C and D). Similar to *Cd47* knockdown (Figure 2, J–L), we observed that miR-34a overexpression strongly inhibited CD47 protein levels and promoted tumor phagocytosis by M1 macrophages; cotreatment with AAV-CD47 in mice completely reversed the effect of miR-34a (Figure 4, D–G, and Supplemental Figure 10, E–G). These results indicated that the escape from innate immune surveillance induced by CD47 was controlled, at least in part, by miR-34a.

### The PI3K/STAT3 axis mediates KRAS-driven miR-34a suppression and CD47 activation.

**Figure 5:**
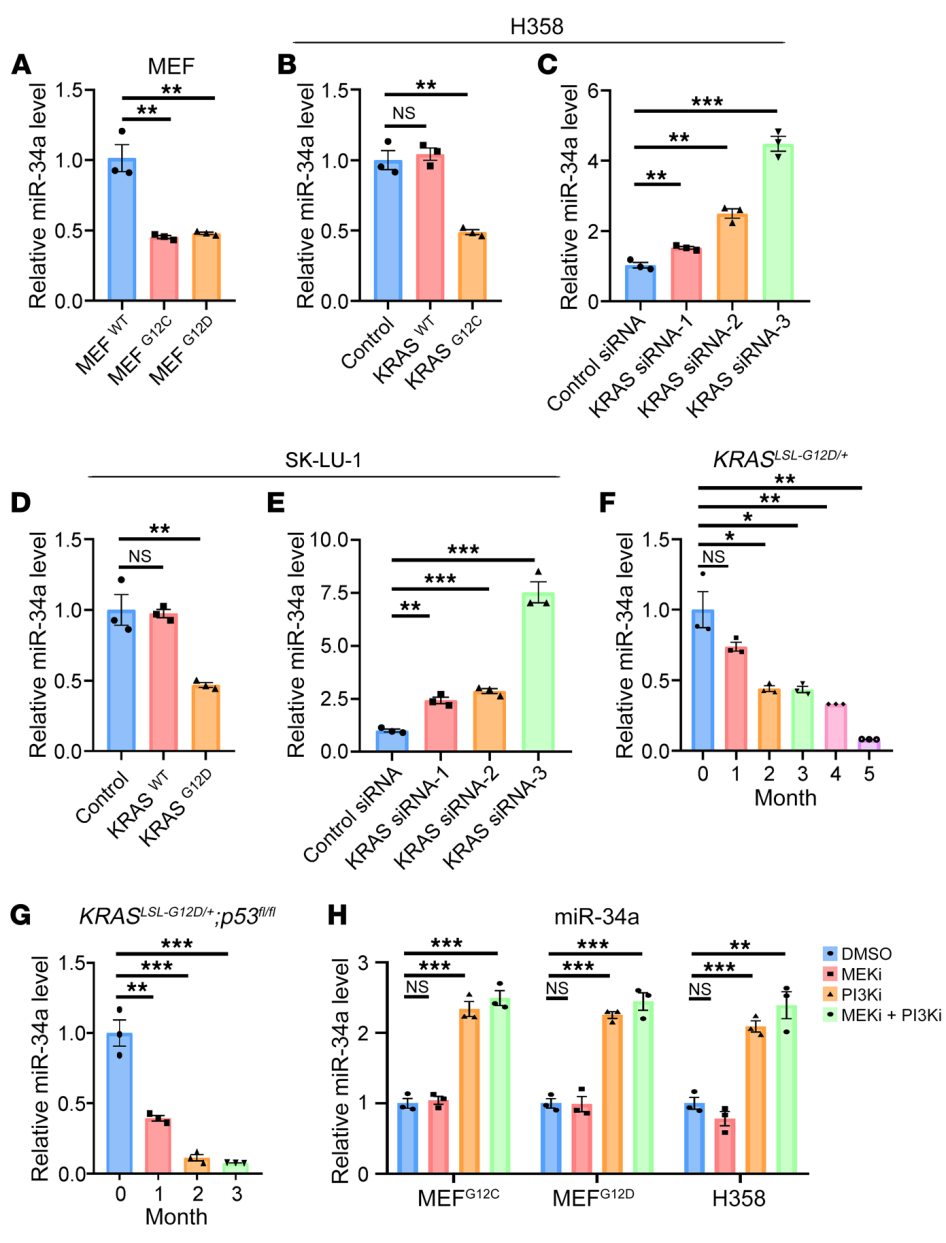
KRAS signaling modulates miR-34a expression in lung cancer cell lines and animal models. (**A**–**E**) Quantitative RT-PCR analysis of the effect of *Kras* mutation status on miR-34a expression levels in MEFs (**A**); H358 cells overexpressing KRAS^WT^ or KRAS^G12C^ (**B**); H358 cells transfected with 3 *KRAS* siRNAs (**C**); SK-LU-1 cells overexpressing KRAS^WT^ or KRAS^G12D^ (**D**); and SK-LU-1 cells transfected with 3 *KRAS* siRNAs (**E**) (*n* = 3). (**F** and **G**) Quantitative RT-PCR analysis of the relative expression levels of miR-34a in whole-lung extracts from *Kras^LSL-G12D/+^* or *Kras^LSL-G12D/+^ p53^fl/fl^* mice at different time points (*n* = 3 mice). (**H**) Effect of MEK and PI3K inhibition on the expression of miR-34a. MEF^G12C^, MEF^G12C^, or H358 cells were treated with DMSO, an MEK inhibitor (MEKi), a PI3K inhibitor (PI3Ki) or a combination of both inhibitors. Relative miR-34a expression levels were determined by quantitative RT-PCR (*n* = 3). Data are shown as the mean ± SEM. *P < 0.05, **P < 0.01, and ***P < 0.001, by 1-way ANOVA (**A**–**H**).

To illustrate how miR-34a connects oncogenic KRAS signaling to CD47 expression, we first determined the relationship between *KRAS* mutation and expression and miR-34a expression. In *Ras*-less MEFs, overexpression of KRAS^G12C^ or KRAS^G12D^ decreased miR-34a levels compared with overexpression of KRAS^WT^ (Figure 5A). Similarly, overexpression of KRAS^G12C^ or KRAS^G12D^, but not KRAS^WT^, decreased miR-34a levels in H358 and SK-LU-1 cells (Figure 5, B and D). In contrast, KRAS knockdown resulted in an increase in miR-34a expression in both cell lines (Figure 5, C and E). A gradual decrease in miR-34a levels was also observed in both the *KRAS^LSL-G12D/+^* and *KRAS^LSL-G12D/+^* *p53^fl/fl^* mice upon activation of the oncogenic activity of KRAS^G12D^ (Figure 5, F and G). These data indicated that oncogenic *KRAS* mutations functioned as a negative regulator of miR-34a expression during *KRAS*-driven lung tumorigenesis.

**Figure 6:**
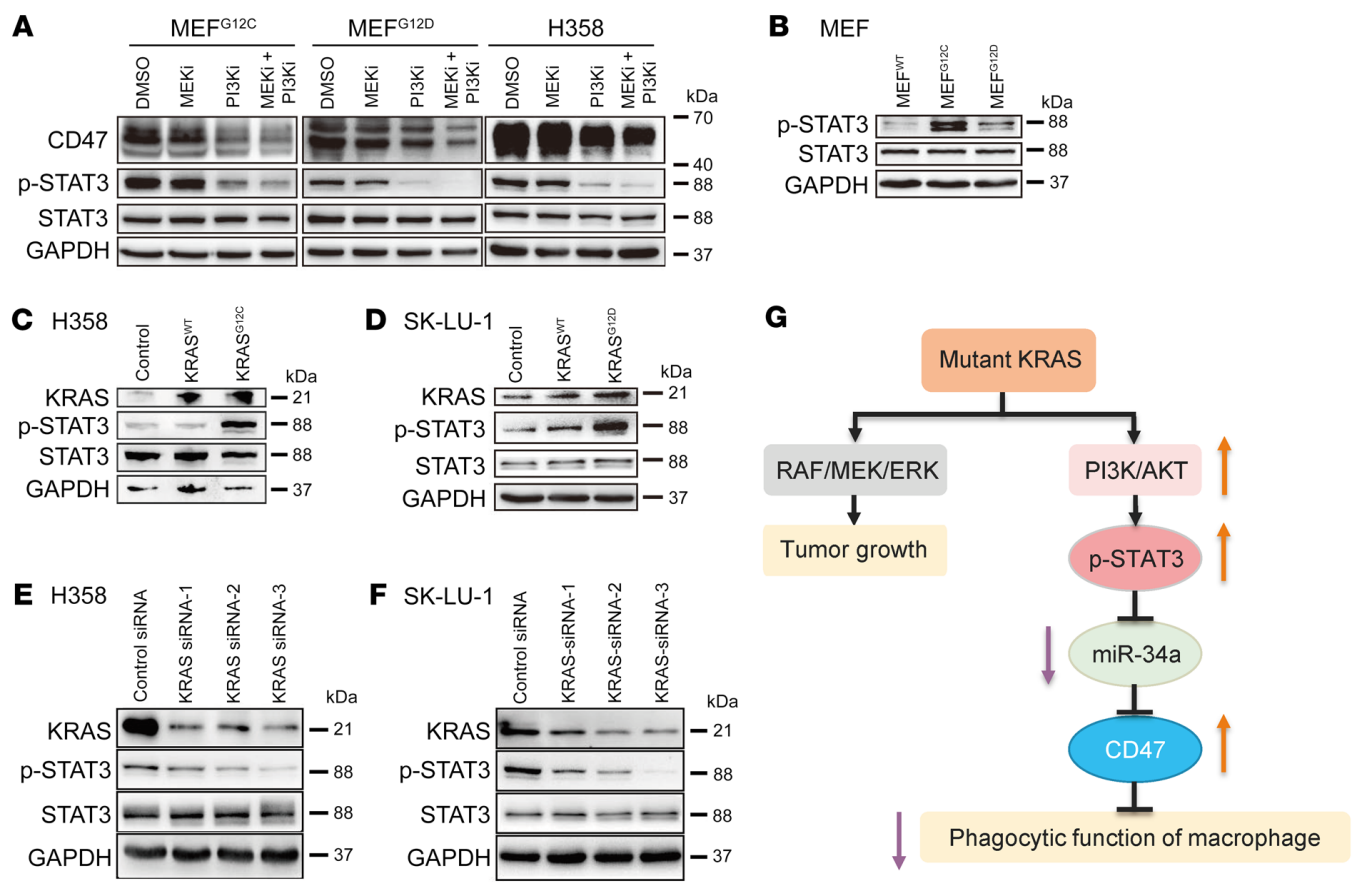
KRAS modulates CD47 expression through the PI3K/STAT3/miR-34a signaling axis. (**A**) Effect of MEK and PI3K inhibition on the expression of CD47, p-STAT3, and total STAT3 in MEFs and H358 cells. (**B**–**F**) Western blot analysis of the expression levels of KRAS, p-STAT3, and total STAT3 in MEFs (**B**); H358 cells overexpressing KRAS^WT^ or KRAS^G12C^ (**C**), SK-LU-1 cells overexpressing KRAS^WT^ or KRAS^G12D^ (**D**), H358 cells transfected with 3 *KRAS* siRNAs (**E**), or SK-LU-1 cells transfected with 3 *KRAS* siRNAs (**F**). (**G**) Schematic of the signaling pathways involved in the regulation of CD47 expression and macrophage phagocytic function by *KRAS* mutation.

To determine which signaling pathway downstream of KRAS is responsible for regulating miR-34a expression, we blocked the RAF/MEK/ERK pathway with a MEK inhibitor (GSK1120212, trametinib) and the PI3K/AKT pathway with a PI3K inhibitor (GDC-0941, pictilisib), respectively, in MEFs and H358 cells. While the MEK inhibitor had no effect on the expression of miR-34a and CD47, the PI3K inhibitor, either alone or in combination with the MEK inhibitor, substantially induced miR-34a expression (Figure 5H) and decreased CD47 levels (Figure 6A and Supplemental Figure 11A) in both MEFs and H358 cells. These results revealed a direct mechanistic link between the PI3K pathway and miR-34a expression in lung cancer cells.

Recent studies have uncovered an interdependence of PI3K and STAT3 signaling in cancer cells (25, 26); in particular, STAT3 was phosphorylated at Tyr705 and activated in a PI3K-dependent manner. Because STAT3 is a well-known transcriptional repressor of miR-34a that negatively controls the expression of miR-34a via a conserved STAT3-binding site in the first intron of the *MIR34A* gene (27), we speculated that KRAS might regulate miR-34a expression through PI3K/STAT3 signaling. To prove this hypothesis, we first measured STAT3 phosphorylation (p-STAT3) levels in MEF cells and found that the p-STAT3 levels were higher in MEF^G12C^ and MEF^G12D^ cells than in MEF^WT^ cells (Figure 6B and Supplemental Figure 11, B and C). Moreover, overexpression of KRAS^G12C^ or KRAS^G12D^ , but not KRAS^WT^, increased the p-STAT3 levels in H358 and SK-LU-1 cells (Figure 6, C and D, and Supplemental Figure 11, D–I). In contrast, KRAS knockdown decreased p-STAT3 levels in both cell lines (Figure 6, E and F, and Supplemental Figure 11, J–O). We then determined the impact of MEK and PI3K inhibitors on STAT3 phosphorylation. Similar to the effect on miR-34a, the PI3K inhibitor, but not the MEK inhibitor, suppressed KRAS-driven STAT3 phosphorylation in both MEFs and H358 cells (Figure 6A and Supplemental Figure 11, P and Q). Likewise, treatment with a STAT3 inhibitor (Stattic) also caused sustained inhibition of STAT3 activation (Tyr705 phosphorylation) and CD47 expression as well as elevation of miR-34a in H358 cells (Supplemental Figure 12). Taken together, these data suggested that KRAS signaling can suppress miR-34a expression via the PI3K/STAT3 axis, which in turn relieves miR-34a–dependent repression of CD47, leading to escape from innate immune surveillance and tumor progression (Figure 6G).

### KRAS mutation status is positively correlated with CD47 expression in several lung adenocarcinoma cohorts.

**Figure 7:**
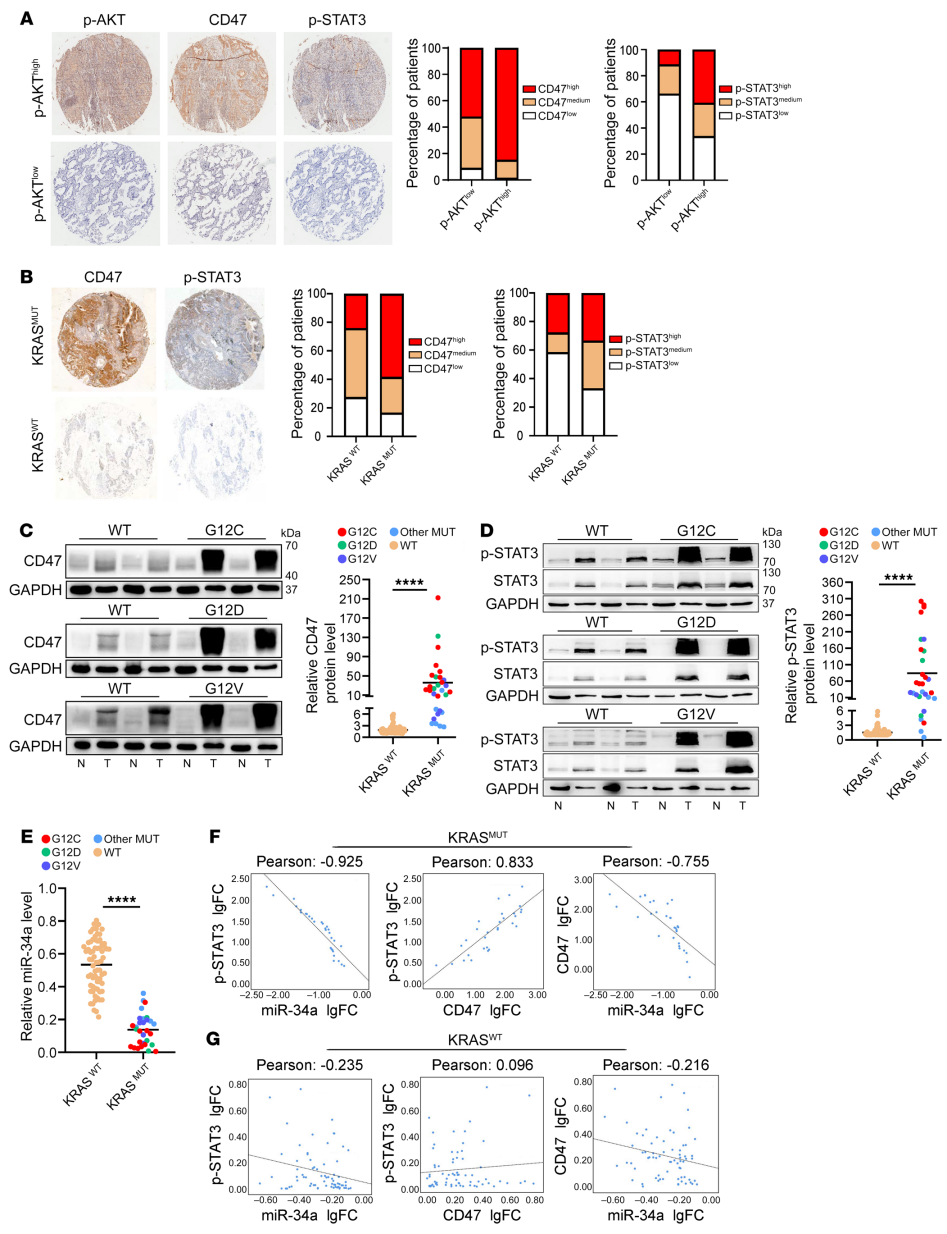
Clinical relevance of *KRAS* mutation status and CD47 expression in patients with lung adenocarcinoma. (**A**) Correlation analysis of KRAS activity and CD47 expression in the first patient cohort consisting of 157 lung adenocarcinoma samples. IHC staining was performed to analyze CD47 and p-STAT3 levels in the tissue microarray chips stratified by high or low KRAS activity (measured by p-AKT levels because of the lack of *KRAS* mutation information). Representative IHC images are shown. Original magnification, ×4. Bar graphs show CD47 and p-STAT3 expression in patients with low and high p-AKT levels (*n* = 98 and *n* = 59, respectively). CD47 and p-STAT3 expression status was stratified on the basis of IHC scores. (**B**) IHC analysis of CD47 and p-STAT3 expression in an in-house–generated tissue microarray containing 40 lung adenocarcinoma samples (28 *KRAS^WT^* and 12 *KRAS^MUT^*). Representative IHC images are shown. Original magnification, ×4. Bar graphs show CD47 and p-STAT3 expression levels in *KRAS^WT^* and *KRAS^MUT^* tumors (*n* = 28 and *n* = 12, respectively). CD47 and p-STAT3 expression status was stratified on the basis of IHC scores. (**C**–**E**) Correlation analysis of *KRAS* mutation status and the expression of CD47, p-STAT3, and miR-34a in the third lung adenocarcinoma patient cohort, with the *KRAS* mutation status determined by deep sequencing. (**C**) Immunoblot analysis of CD47 expression in *KRAS^WT^*, *KRAS^G12C^*, *KRAS^G12D^*, and *KRAS^G12V^* patients. Representative blots are shown along with quantitative analysis (*n* = 70 *KRAS^WT^* and *n* = 30 *KRAS^MUT^*, respectively). (**D**) Immunoblot analysis of p-STAT3 expression in samples from *KRAS^WT^* and *KRAS^MUT^* patients. (**E**) Quantitative RT-PCR analysis of miR-34a levels in samples from *KRAS^WT^* and *KRAS^MUT^* patients. (**F** and **G**) Pearson’s correlation coefficient analysis of correlations between expression levels of p-STAT3, miR-34a, and CD47 in the *KRAS^MUT^* (**F**) and *KRAS^WT^* (**G**) lung adenocarcinoma samples. Data are shown as the mean ± SEM. ****P < 0.0001, by unpaired *t* test (**C**–**E**) or Pearson’s correlation test (**F** and **G**). N, normal; T, tumor; FC, fold change.

To further explore the clinical relevance of our findings, we assessed the correlation of *KRAS* mutation status with CD47 expression in 3 independent lung adenocarcinoma cohorts. The first cohort was a commercial tissue microarray containing 157 pairs of lung adenocarcinoma and normal adjacent tissue samples, which lacked *KRAS* mutation information. We performed IHC analysis of CD47, p-STAT3, and p-AKT and found that CD47 levels were consistently higher in the tumor tissues than in the normal control tissues (Supplemental Figure 13A). High CD47 expression was also positively correlated with advanced tumor grade and poor survival (Supplemental Figure 13, B and C). To evaluate the correlation of CD47 expression with KRAS signaling, we used the p-AKT level (downstream effector of PI3K) as a readout for KRAS activity and segregated all patient samples into high and low p-AKT groups. As expected, both CD47 and p-STAT3 levels were higher in the high p-AKT group than in the low p-AKT group (Figure 7A). Moreover, while high p-AKT expression was positively correlated with poor overall survival in patients with lung adenocarcinoma, coordinated activation of KRAS (p-AKT^hi^) and CD47 further increased the probability of a poor prognosis (Supplemental Figure 13, D and E; HR, 1.81 vs. 1.46).

In the second cohort, we performed IHC analysis of CD47 and p-STAT3 expression in an in-house–generated tissue microarray containing paired tumor samples and adjacent normal tissue samples from 12 patients with *KRAS^MUT^* lung adenocarcinoma and 28 patients with *KRAS^WT^* lung adenocarcinoma. We determined the *KRAS* mutation status by deep sequencing. In both *KRAS^MUT^* and *KRAS^WT^* patients, we found that CD47 was highly expressed in the tumor samples compared with their normal counterpart samples (Supplemental Figure 13F). Compared with *KRAS^WT^* tumors, the tumor samples with *KRAS* mutations displayed higher expression of CD47 and p-STAT3 (Figure 7B).

In the third cohort, we determined the *KRAS* mutation status and the expression levels of CD47, p-STAT3, and miR-34a in 100 pairs of lung adenocarcinoma and normal tissue samples. Thirty lung adenocarcinoma samples were confirmed to be *KRAS*-mutant and seventy were WT. CD47 and p-STAT3 protein levels were consistently upregulated, and miR-34a was downregulated in the lung adenocarcinoma samples compared with the paired normal controls, regardless of *KRAS* mutation status (Supplemental Figure 13, G–I). In the tumor samples, CD47 and p-STAT3 expression levels were much higher in the *KRAS^MUT^* patients than in the *KRAS^WT^* patients (Figure 7, C and D). The opposite trend was observed for miR-34a (Figure 7E). The tight correlation between *KRAS* mutation status and CD47, p-STAT3, and miR-34a expression levels was further demonstrated by Pearson’s correlation coefficient analysis, in which a reciprocal expression pattern between p-STAT3 and miR-34a and between CD47 and miR-34a, as well as a coincident pattern between p-STAT3 and CD47, were observed in *KRAS^MUT^* patients but not in *KRAS^WT^* patients (Figure 7, F and G). In summary, these results from 3 independent lung adenocarcinoma cohorts were consistent with each other and confirmed that *KRAS* mutation status is positively correlated with CD47 expression in patients with lung adenocarcinoma.

### Targeted therapy against KRAS^G12C^ inhibits CD47 signaling and restores innate immune surveillance in animal models of lung cancer.

**Figure 8:**
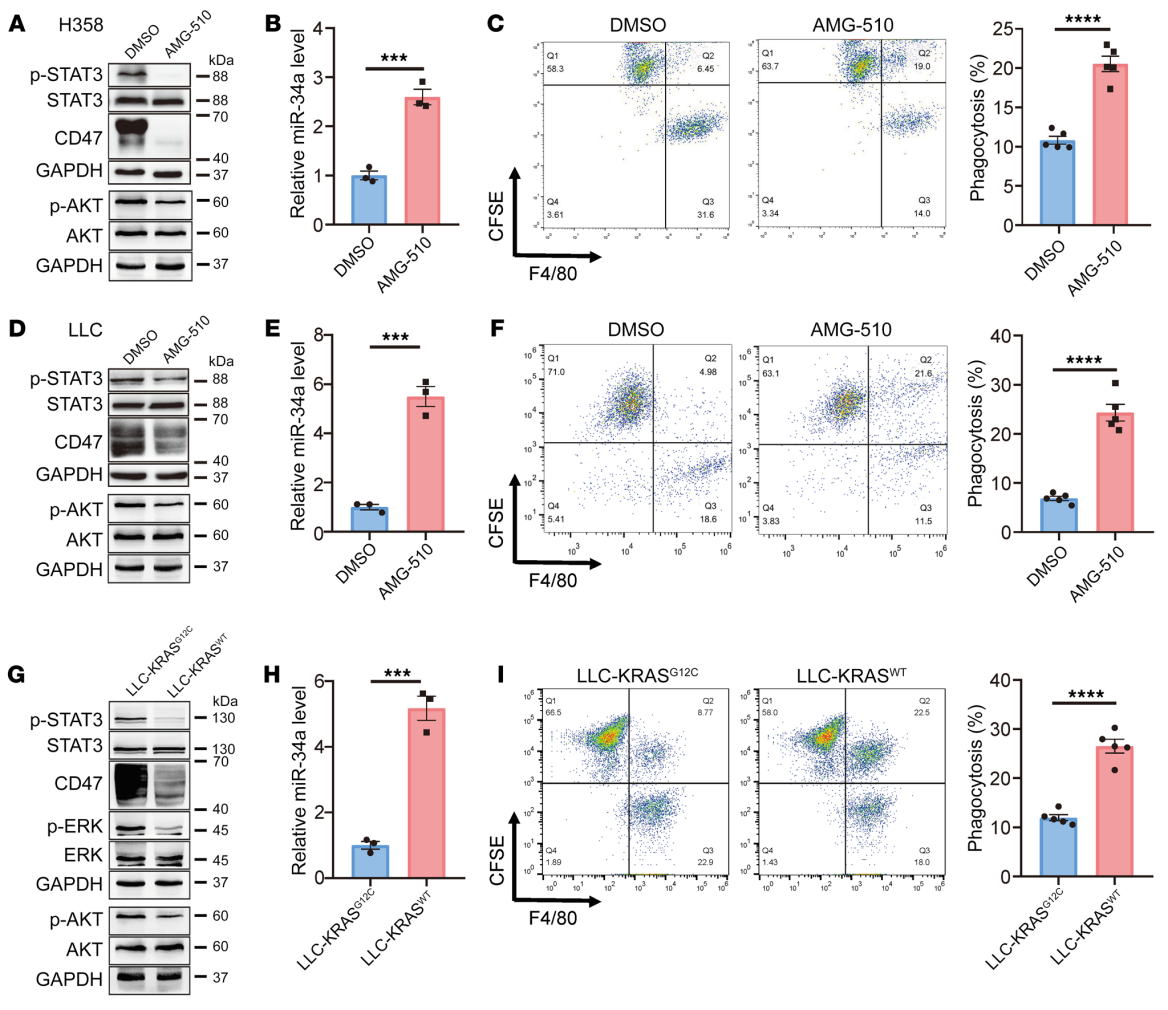
The KRAS^G12C^ inhibitor AMG 510 inhibits CD47 signaling and promotes macrophage phagocytosis of tumor cells in vitro. (**A**–**C**) AMG 510 treatment rendered H358 cells sensitive to phagocytosis by macrophages. (**A** and **B**) Effect of AMG 510 treatment (24 h) on expression levels of p-STAT3, p-AKT, CD47, and miR-34a in H358 cells. (**C**) KRAS^G12C^ inhibition increased the phagocytosis of H358 cells by macrophages. Cells were treated with AMG 510 for 24 hours before coculturing with human peripheral blood monocyte–derived macrophages. Phagocytosis of H358 cells by macrophages was analyzed by flow cytometry. Representative FACS plots and quantification (*n* = 5) are shown. (**D**–**F**) AMG 510 treatment rendered LLC cells sensitive to phagocytosis by macrophages. Experiments identical to those in **A**–**C** were performed. (**G**–**I**) Effect of *Kras* mutation status on CD47 expression and macrophage phagocytosis in LLC cells. LLC cells (heterozygous for *Kras^G12C^*) were converted to *Kras^WT^* using CRISPR/Cas9. Experiments similar to those in **A**–**C** were then carried out for the cell pair. Data are shown as the mean ± SEM. ***P < 0.001 and ****P < 0.0001, by unpaired *t* test (**B**, **C**, **E**, **F**, **H**, and **I**).

From a translational perspective, the link between *KRAS* mutations and innate immune evasion suggests that targeting the KRAS/CD47 axis might compromise the ability of cancer cells to evade innate immune surveillance and increase their susceptibility to macrophage phagocytosis. Consistent with our hypothesis, treatment of the H358 human lung cancer cell line or the Lewis lung carcinoma (LLC) (heterozygous for *Kras^G12C^*) mouse lung cancer cell line with the FDA-approved KRAS^G12C^ inhibitor AMG 510 (28) attenuated KRAS activity and p-STAT3 and CD47 expression, enhanced miR-34a expression, and stimulated macrophage-mediated phagocytosis in both H358 and LLC cells (Figure 8, A–F, and Supplemental Figure 14, A–C). In addition, the PI3K agonist 740 Y-P, but not the MAPK agonist PAF C-16, partially reversed the inhibitory effect of AMG 510 on p-STAT3 and CD47 expression, further confirming the involvement of PI3K in KRAS regulation of STAT3 and CD47 (Supplemental Figure 15). Moreover, similar to the pharmacological inhibitors, when we used CRISPR/Cas9 to convert the genotype of the LLC mouse lung cancer cell line from *Kras^G12C^* to *Kras^WT^* and compared the phenotype between the isogenic cell pair LLC-KRAS^G12C^ and LLC-KRAS^WT^, the latter showed much lower levels of KRAS activity and CD47 expression but much higher miR-34a and macrophage-mediated phagocytosis (Figure 8, G–I, and Supplemental Figure 14, D and E).

**Figure 9:**
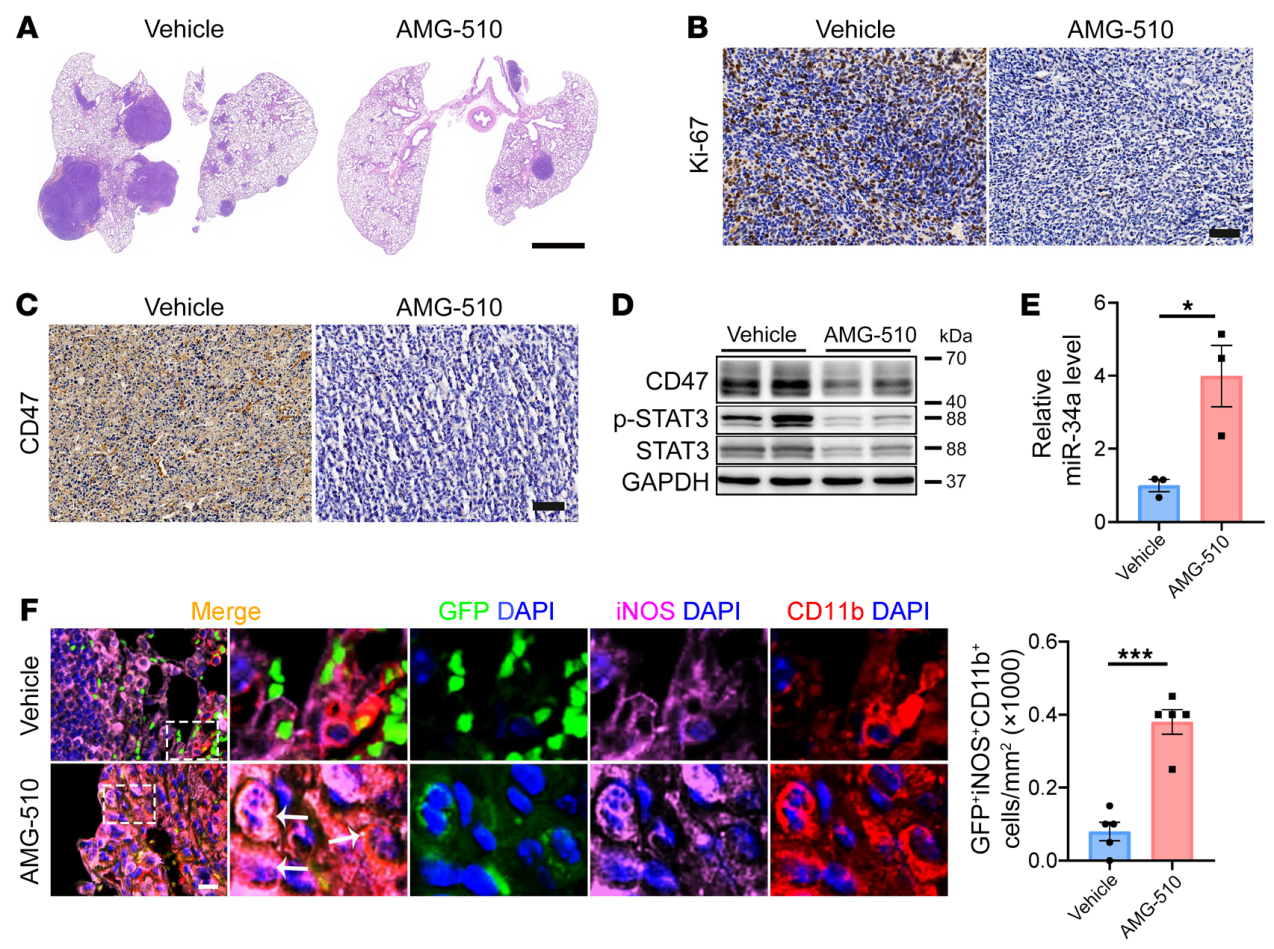
The KRAS^G12C^ inhibitor AMG 510 inhibits CD47 signaling and promotes macrophage phagocytosis of tumor cells in vivo. EGFP-labeled LLC cells were injected via the tail vein into C57BL/6 mice. After tumor formation, mice were administered AMG 510 via oral gavage once a day for 8 days. (**A**) Representative H&E-stained lung sections. Scale bar: 2 mm. (**B**) Representative Ki-67 staining of lung sections. Scale bar: 20 μm. (**C**) Representative CD47 staining of lung sections. Scale bar: 20 μm. (**D**) Representative immunoblots of CD47 and p-STAT3 in lung tumor cells. (**E**) Quantitative RT-PCR analysis of miR-34a expression in lung tumors (*n* = 3). (**F**) Immunofluorescence staining for CD11b (red), iNOS (purple), and DAPI (blue) in lung tumors showing an increase in macrophage phagocytosis of tumor cells with AMG 510 treatment. Representative images and quantification results (*n* = 5 mice) are shown. Arrows indicate GFP^+^iNOS^+^CD11b^+^ cells. Scale bar: 50 μm. Original magnification, ×40 (enlarged insets). Data are shown as the mean ± SEM. *P < 0.05 and ***P < 0.001, by unpaired *t* test (**E** and **F**).

Finally, we tested the effect of AMG 510 on the tumor immune microenvironment in vivo. The LLC mouse lung cancer cells were injected via the tail vein into immunocompetent C57BL/6 mice to establish a lung colonization model. AMG 510 treatment for 8 days significantly suppressed tumor growth, inhibited KRAS activity, STAT3 phosphorylation, and CD47 expression, and stimulated miR-34a expression in tumor tissues (Figure 9, A–E, and Supplemental Figure 16, A–E). Most importantly, AMG 510 treatment significantly increased the infiltration of M1 macrophages into the tumor tissue as well as tumor phagocytosis by the M1 macrophages (Figure 9F and Supplemental Figure 16F). Taken together, these data proved that the in vivo antitumor effect of KRAS^G12C^ inhibitors might, at least in part, be due to the reactivation of the innate immune response to cancer cells.

## Discussion

Oncogenic *KRAS* mutations induce activation of the RAF/MEK/ERK and PI3K/AKT signaling pathways in a cell-autonomous manner, leading to constitutive activation of cell proliferation and inhibition of cell death; however, the role of KRAS signaling in the tumor microenvironment and tumor immune response is poorly understood. Our findings provide the first evidence, to our knowledge, that *KRAS* mutations could suppress the innate antitumor immune response by regulating CD47 expression in lung cancer cells. Thus, mutant KRAS communicates with macrophages via the CD47/SIRPα axis and renders tumor cells insensitive to phagocytosis by macrophages. Interestingly, 2 recent findings by Coelho et al. (12) and Canon et al. (13) demonstrated that *KRAS* mutations could also restrict the adaptive immune response through upregulation of PD-L1 and induce monoresistance to T cells. These findings complement each other and together indicate that mutant *KRAS* lies at the heart of tumor immune evasion, with oncogenic KRAS signaling functioning as a 2-pronged approach to restrict the antitumor potential of both innate and adaptive immune systems. Inhibition of KRAS signaling could therefore render cancer cells more susceptible to immune attack by both T cells and macrophages, which might contribute to the overall antitumor effect of KRAS inhibitors in vivo. It is also reasonable to speculate that the combination of KRAS inhibitors and immune checkpoint inhibitors, including inhibitors of PD-1/PD-L1 or CD47/SIRPα, could provide synergistic benefits to patients with *KRAS*-driven cancers.

Disruption of the CD47/SIRPα axis using anti-CD47 mAbs has demonstrated efficacy in various preclinical models and has already entered phase I/II clinical trials (29, 30). However, 2 major obstacles may delay the clinical translation of anti-CD47 antibodies in cancer immunotherapy. First, predictive biomarkers of therapeutic response are underdeveloped. In this study, we established a positive correlation between *KRAS* mutation status and CD47 overexpression in patients with lung adenocarcinoma. Thus, patients with *KRAS*-mutant lung adenocarcinoma might be especially sensitive to anti-CD47 immunotherapy. Second, given the ubiquitous expression of CD47 in normal tissues, anti-CD47 antibodies may elicit severe side effects such as anemia, thrombocytopenia, and leukopenia, as observed in animal models (31, 32). The combination with KRAS inhibitors might help reduce the effective dose of anti-CD47 antibodies, thereby limiting the side effects and toxicities associated with anti-CD47 therapy. Altogether, a better understanding of how the KRAS/CD47 axis evades innate immune surveillance may provide a framework for patient selection and combination therapies to enhance the effectiveness of anti-CD47 immunotherapy.

Taken together, this study demonstrates that active KRAS can promote innate immune evasion of lung cancer through upregulation of CD47. These findings not only extend our understanding of the role of KRAS signaling in tumor immune surveillance but could also be exploited for the treatment of *KRAS*-driven cancers.

## Methods

### Study design.

For the *KRAS*-driven spontaneous lung cancer model, *Kras^LSL-G12D/+^* and *Kras^LSL-G12D/+^* *p53^fl/fl^* transgenic mice were intratracheally administered adeno-Cre to induce pulmonary adenocarcinoma formation. Tumor growth, CD47 expression, and macrophage infiltration were assessed at different time points or were evaluated when disrupting the KRAS/CD47 signaling axis. For the animal models of lung cancer, C57BL/6 mice were injected via the tail vein with LLC cells (*Kras^G12C^*) and administered the KRAS^G12C^ inhibitor AMG 510 by oral gavage after tumor formation; then, tumor regression, CD47 expression, and macrophage infiltration were assessed. For determination of the molecular mechanism underlying KRAS-mediated CD47 activation, a *Ras*-less MEF model stably overexpressing KRAS^G12C^, KRAS^G12D^, or KRAS^WT^ and the human lung cancer cell lines H358 (*KRAS^G12C^*) and SK-LU-1 (*KRAS^G12D^*) were cultured and assessed. For the in vitro phagocytosis assay, FACS and fluorescence microscopy were performed to analyze the phagocytosis of primary lung tumor cells or lung cancer cell lines by human peripheral blood monocyte–derived macrophages. For analysis of lung adenocarcinoma patient samples, the correlation of *KRAS* mutation status with CD47 expression was assessed in 3 independent lung adenocarcinoma cohorts.

### Reagents.

AMG 510, the MEK inhibitor GSK1120212 (trametinib), the PI3K inhibitor GDC-0941 (pictilisib), and the PI3K agonist 740 Y-P (HY-P0175) were purchased from MedChemExpress, the STAT3 inhibitor Stattic was purchased from Selleck, and the MAPK agonist PAF C-16 (sc-201009) was purchased from Santa Cruz Biotechnology. Unless otherwise noted, all chemicals were purchased from MilliporeSigma.

### Cell culture.

The human lung cancer cell lines H358 and SK-LU-1 were obtained from the American Type Culture Collection (ATCC). The human embryonic kidney (HEK) cell line HEK293T and the mouse lung LLC cell line were obtained from the Shanghai Institute of Cell Biology, Chinese Academy of Sciences (Shanghai, China). Cells were certified by short tandem repeat (STR) analysis and regularly checked for mycoplasma contamination. *Ras*-less MEF cell lines overexpressing different KRAS mutations were obtained from the NIH RAS Initiative and cultured as indicated in https://www.cancer.gov/research/key-initiatives/ras/ras-central/blog/2017/rasless-mefs-drug-screens (33). H358 cells were maintained in RPMI 1640 medium (C11875500BT, Gibco, Thermo Fisher Scientific) supplemented with 10% FBS (10099-141, Gibco, Thermo Fisher Scientific), and SK-LU-1, LLC, and HEK293T cells were maintained in high-glucose (4.5 g/L) DMEM (C11995500BT, Gibco, Thermo Fisher Scientific) supplemented with 10% FBS (Gibco, Thermo Fisher Scientific). All cells were incubated in 5% CO_2_ at 37°C in a humidified atmosphere.

### Construction of an LLC-Kras^WT^ cell line using CRISPR/Cas9.

A *Kras* gene–modified LLC cell line (LLC-Kras^WT^) was developed using the GenCRISPR gene-editing kit from GenScript. Briefly, based on the genomic sequences of the parental LLC cell line harboring a heterozygous *Kras^G12C^* mutation (LLC-Kras^G12C^), several guide RNA (gRNA) sequences (best one: GACTGAGTATAAACTTGTGG) were designed to target the area near the *Kras^G12C^* site, and a donor template was designed containing the designated mutation. By transient cotransfection of plasmids carrying the gRNA-Cas9 plasmid and donor plasmid, the *Kras* gene was targeted and mutated from G12C to WT. Single clones with successful gene conversion were selected by limiting dilution and expansion in 96-well plates and verified by Sanger sequencing.

### Patient tissue samples.

A total of 4 separate patient cohorts were used in this study. For the assessment of macrophage phagocytosis and CD47 expression in fresh human lung adenocarcinoma tissues, 18 pairs of tumor and normal adjacent tissue samples were collected from patients with lung adenocarcinoma who were undergoing surgery at the Jiangsu Cancer Hospital. Briefly, tissues were placed in 1.0 mL RPMI 1640 with Liberase TL (0.2 mg/mL; Roche) and DNase I (20 μg/mL; Ambion) and minced with scissors into sub-millimeter pieces. Tissues were then dissociated into single cells using the gentleMACS program (Miltenyi Biotec) at 37°C for 40 minutes, according to the manufacturer’s instructions. Cells were then passed through a 70 mm mesh and centrifuged at 350*g* for 5 minutes. Cell pellets were resuspended, and 1 aliquot of the cells (1 × 10^6^) was incubated with 1 mg fluorescently conjugated mAbs against human CD47 (BD Biosciences) or the isotype control. Another aliquot of the cells was subjected to the in vitro phagocytosis assay. Samples were fixed in 4% paraformaldehyde, washed, resuspended in FACS buffer, and analyzed by flow cytometry using a FACScalibur flow cytometer (BD Biosciences).

Samples from 3 patient cohorts were used for the correlation analysis of *KRAS* mutation and CD47 levels. The first cohort was a commercial tissue microarray containing 157 pairs of lung adenocarcinoma and normal adjacent tissue samples purchased from Shanghai Outdo Biotech. The second cohort was an in-house–generated tissue microarray containing 12 pairs of *KRAS^MUT^* lung adenocarcinoma and adjacent normal tissue samples and 28 pairs of *KRAS^WT^* lung adenocarcinoma and adjacent normal tissue samples; these samples were obtained from the Jiangsu Biobank of Clinical Resources (located at Jiangsu Cancer Hospital, Nanjing, China). The third cohort consisted of 100 pairs of lung adenocarcinoma and normal adjacent tissue samples obtained from the Jiangsu Biobank of Clinical Resources. These samples were selected on the basis of a clear pathological diagnosis. Approximately 5 g segments of lung adenocarcinoma and normal tissues were promptly transferred to containers with liquid nitrogen and frozen at –80°C. The *KRAS* mutation status in these samples was determined by TA cloning and sequencing of RT-PCR products. Patient information is shown in Supplemental Table 2.

### Genetic models of lung cancer.

The *Kras^LSL-G12D/+^* and *Kras^LSL-G12D/+^* *p53^fl/fl^* transgenic mice were provided by Hongbin Ji (Shanghai Institutes for Biological Sciences). The mice were maintained on a 12 hour light/12-hour dark cycle (lights on at 7 am) with free access to food and water. For *KRAS^G12D^* activation in mouse lungs, 6-week-old *Kras^LSL-G12D/+^* and *Kras^LSL-G12D/+^* *p53^fl/fl^* mice were first anesthetized with sodium pentobarbital, and then 5 × 10^6^ PFU adeno-Cre were diluted with PBS to obtain a final volume of 50 μL and given through intratracheal administration (21, 22). At different time points after adeno-Cre administration (0 and 5 months for *Kras^LSL-G12D/+^* mice and 0, 1, and 3 months for *Kras^LSL-G12D/+^* *p53^fl/fl^* mice), the mice were anesthetized to evaluate tumor growth by micro-CT scanning or euthanized to confirm lung adenocarcinoma formation by histological analysis. Histological analysis was performed by H&E staining. Excised lung adenocarcinomas were also processed to determine CD47 expression, macrophage infiltration by Western blotting, immunofluorescence staining, or IHC analyses.

For AAV-mediated silencing of *Cd47*, an shRNA of *Cd47* was subcloned into the AAV vector AAV9-CAG-EGFP (Sunbio) (AAV-*Cd47* shRNA). An AAV encoding scrambled shRNA (AAV-control shRNA) served as the negative control. *Kras^LSL-G12D/+^* *p53^fl/fl^* mice were coadministered intratracheally with adeno-Cre along with the AAV-control shRNA or AAV-*Cd47* shRNA. Then, the mice were divided into 2 groups and monitored to determine either survival time or tumor regression. For survival analysis, the mice were monitored for 150 days without any further treatment. For tumor size, the mice were anesthetized to evaluate tumor growth by micro-CT scanning or euthanized to confirm lung adenocarcinoma formation by histological analysis at 90 days. Excised lung adenocarcinomas were also processed to determine CD47 expression and macrophage infiltration by Western blotting, immunofluorescence staining, or IHC analysis.

For the overexpression of miR-34a in mice, an AAV9-CAG-EGFP (Sunbio) encoding miR-34a (AAV–miR-34a) was used, with or without simultaneous administration of an AAV9-CAG-EGFP (Sunbio) expressing the CD47 ORF (AAV-CD47). An AAV encoding scrambled RNA (AAV-scrRNA) served as the negative control for AAV–miR-34a, and an AAV that did not express a transgene (AAV control) served as the negative control for AAV-CD47. *Kras^LSL-G12D/+^* *p53^fl/fl^* mice were intratracheally coadministered adeno-Cre along with the combination of AAV scrRNA plus AAV-control, AAV–miR-34a plus AAV-control, or AAV–miR-34a plus AAV-CD47. The mice were then assessed as described above.

### Animal models of lung cancer.

To generate a lung colonization model of human lung cancer, 5 × 10^6^ H358 cells stably transfected with EGFP were intravenously injected into BALB/c nude mice via the tail vein. After 3 weeks, 1 mouse was euthanized every week to ensure successful lung tumor formation, as assessed by immunofluorescence. Then, the tumor-bearing mice were divided into 3 groups and monitored to determine macrophage infiltration, as assessed by immunofluorescence at different times.

To generate a lung colonization model of lung cancer in immune-competent mice, 5 × 10^6^ LLC cells were intravenously injected into C57BL/6 mice via the tail vein. After 15 days, the mice were monitored using noninvasive micro-CT scanning to ensure successful tumor formation in the lungs. Then, the tumor-bearing mice were randomly divided into 2 groups and were orally administered 100 mg/kg AMG 510 or vehicle control. After 8 days, the mice were euthanized to evaluate lung tumor burden by histopathological staining. Excised lung tumors were also processed to determine CD47 expression and macrophage infiltration by Western blotting, immunofluorescence staining, or IHC analyses. Moreover, single-cell suspensions of tumors were prepared for flow cytometry as described previously (13). Briefly, tumors were placed in 1.0 mL RPMI 1640 with Liberase TL (0.2 mg/mL; Roche) and DNase I (20 μg/mL; Ambion) and minced with scissors to sub-millimeter-sized pieces. Tissues were homogenized in the MACS tissue homogenizer using the gentleMACS program according to the manufacturer’s instructions and then incubated at 37°C for 40 minutes. Specimens were passed through a 70 mm mesh and centrifuged at 350*g* for 5 minutes. Cell pellets were resuspended, and cell labeling was performed by incubating 1 × 10^6^ cells with 0.5 μg fluorescently conjugated antibodies directed against mouse F4/80 (BD Biosciences). Intracellular iNOS antibody (BD Biosciences) staining was performed following the intracellular staining protocol. Samples were fixed in 4% paraformaldehyde, washed, resuspended in FACS buffer, and analyzed by flow cytometry (FACScalibur, BD Biosciences).

### Micro-CT scanning.

Micro-CT analysis was performed to assess lung tumor growth because the micro-CT images clearly distinguished the lung tumors from the surrounding tissue even without a contrast agent, and the reconstructed 3D pulmonary images can easily differentiate the tumors from the blood vessels (34). Briefly, micro-CT scans were performed using a SkyScan 1176 micro-CT analyzer, which scanned a 360° area at a resolution of 50 μm with a rotation step of 0.5. The system comprised 2 metallochromic tubes equipped with a fixed 0.5 mm Al filter and two 1,280 × 1,024 pixel digital x-ray cameras. Images were acquired at 60 kV and 134 μA. The mice were scanned while in a supine position. The micro-CT data were batch sorted, processed, and reconstructed as 3D pulmonary images using the N-Recon program (SkyScan) according to the manufacturer’s instructions. The reconstructed data were subsequently imaged using DataViewer (Extron), and the number of tumors and their volumes were calculated using the CTAn program (SkyScan) according to the manufacturer’s instructions.

### Histopathology.

For histopathological examination of *Kras^LSL-G12D/+^* and *Kras^LSL-G12D/+^* *p53^fl/fl^* mice, whole lung lobes were fixed in 4% paraformaldehyde overnight and embedded in paraffin. H&E staining was performed using a standard method. Digitally scanned images of H&E slides were created with the Aperio ScanScope AT2 at ×20 magnification and analyzed with Aperio’s WebScope software. For quantification of the tumor burden, tumor regions were outlined, and the percentage of the tumor area relative to the total lung area was calculated for each mouse. All tumor burdens were assessed in a blinded fashion, and at least 5 mice per group were included in the analyses.

### Immunofluorescence staining.

Excised lung adenocarcinomas were postfixed for 4 hours in 4% PFA and cryoprotected in 20% and 30% sucrose in 1 × PBS at 4°C. For immunofluorescence analysis, the sections were postfixed for 10 minutes in 4% PFA and then washed with 1 × PBS prior to blocking with 5% normal horse serum with 0.25% Triton X-100 in PBS (1 hour). The sections were then incubated with CD11b, iNOS, TNF-α, or KRAS^G12D^ primary antibodies diluted 1:100 in blocking solution overnight. Detailed information on the primary antibodies used can be found in Supplemental Table 3. The following day, the sections were washed with 1× PBS and subsequently incubated in blocking solution containing a secondary antibody for 1 hour. Then, the sections were washed with 1× PBS and placed in DAPI staining solution for 10 minutes. After the sections were washed with 1× PBS, they were examined with a TCS SP8 inverted laser scanning confocal microscope (Leica). Digital images from the microscope were recorded with LAS X Viewer Software (Leica). Cell counts were performed using Image-Pro Plus 6.0 software (Media Cybernetics) in combination with manual scoring to ensure accuracy.

### IHC.

IHC analysis was performed according to standard protocols. Prior to staining, sections from the lung tumors of the *Kras^LSL-G12D/+^* and *Kras^LSL-G12D/+^* *p53^fl/fl^* mice were baked at 60°C for 1 hour, deparaffinized in xylene, and rehydrated through graded ethanol. Antigen retrieval was performed by heating the sections under high pressure in citrate antigen retrieval solution for approximately 5 minutes. The sections were incubated with mAbs against CD47, iNOS, TNF-α, p-AKT, or p-STAT3 for 60 minutes at room temperature. Detailed information on the primary antibodies used are provided in Supplemental Table 3. The immunoreaction was detected by treatment with diaminobenzidine chromogen for 3 minutes. The immunoreaction images were viewed and captured using the NDP.view.2 software program. Protein expression was assessed by 2 experienced pathologists blinded to the clinical data, who performed the first reading independently and then debated any discrepancies until reaching a consensus.

### sRNA sequencing.

sRNA deep sequencing was performed to examine the miRNA profiles in the lung tumors of the *Kras^LSL-G12D/+^* mice. All sRNA library construction and deep sequencing were performed by Novogene. Briefly, sRNA libraries were constructed using the NEBNext Multiplex Small RNA Library Prep Set for Illumina (New England BioLabs [NEB]). After library quality validation, raw data for each sRNA library were generated on the Illumina HiSeq 2500 platform. The clean reads were obtained after data filtration. Precursor and mature miRNA sequences were obtained from miRBase, version 21. To annotate miRNA, clean reads were mapped to known mouse miRNA precursor sequences using bowtie, and only candidates with no more than 1 mismatch and 2 shifts were counted as miRNA matches. Differential analysis was performed using DESeq2. Significance was set at an uncorrected *P* value of less than 0.05 for broad pattern identification. A fold change threshold was set at greater than 2. A volcano plot was generated using the ggplot2 R package, and heatmaps were generated using the pheatmap R package. The raw sequencing data reported here have been deposited in the Genome Sequence Archive (GSA) of the National Genomics Data Center (NGDC), China National Center for Bioinformation/Beijing Institute of Genomics, Chinese Academy of Sciences (GSA accession: CRA008806) and are publicly accessible at https://ngdc.cncb.ac.cn/gsa

### Cell transfection.

The ORF sequences of WT *KRAS* (*KRAS^WT^*), mutant *KRAS^G12C^*, or mutant *KRAS^G12D^* were synthesized by GenScript and inserted into a CMV-EGFP plasmid. A plasmid that did not express a transgene served as the negative control. *KRAS* siRNAs were purchased from GenePharma. An siRNA with a scrambled sequence served as the negative control. miR-34a mimics and antisense strands were purchased from GenePharma. Control mimic and antisense strands designed to express dsRNAs or single-stranded scrRNAs served as negative controls. H358 and SK-LU-1 cells were seeded in 12-well plates, and each well was transfected with 5 μg *KRAS^WT^*, *KRAS^G12C^*, or *KRAS^G12D^* plasmids or 50 pmol miR-34a mimic, miR-34a antisense, or the corresponding negative controls using Lipofectamine 3000 (Invitrogen, Thermo Fisher Scientific) according to the manufacturer’s instructions. Total RNA and protein were isolated 24 or 48 hours after transfection. Sequences of the synthetic siRNAs, miRNA mimics, and antisense strands are listed in Supplemental Table 4.

### RNA isolation and quantitative RT-PCR assay.

Total RNA extraction, reverse transcription, and TaqMan-based real-time PCR were performed as described previously. Briefly, total RNA was extracted from cultured cells and mouse tumors with TRIzol Reagent (Invitrogen, Thermo Fisher Scientific) according to the manufacturer’s instructions.

For quantitative RT-PCR analysis of miRNAs, 100 ng total RNA was reverse transcribed to cDNA using AMV reverse transcriptase (TaKaRa) and stem-loop RT primers (Applied Biosystems). The following reaction conditions were used: 16°C for 30 minutes, 42°C for 30 minutes, and 85°C for 5 minutes. Real-time RT-PCR was performed using TaqMan miRNA probes (Applied Biosystems) on an Applied Biosystems 7300 Sequence Detection System (Applied Biosystems). The reactions were incubated in a 96-well optical plate at 95°C for 10 minutes followed by 40 cycles at 95°C for 15 seconds and 60°C for 1 minute. All reactions were run in triplicate. After the reactions were complete, the Ct values were determined using fixed threshold settings, and the mean Ct was determined from triplicate PCRs. The relative expression of miRNAs was determined using the 2^ΔΔCt^ method, and U6 snRNA served as the internal control.

For mRNA analysis, 1 μg total RNA was reverse transcribed to cDNA using AMV reverse transcriptase (TaKaRa) and oligo dT primer (TaKaRa). The following reaction conditions were used: 16°C for 30 minutes, 42°C for 30 minutes, and 85°C for 5 minutes. Real-time RT-PCR was performed using SYBR Green PCR Master Mix (Invitrogen, Thermo Fisher Scientific) on an Applied Biosystems 7300 Sequence Detection System. The reactions were incubated in a 96-well optical plate at 95°C for 10 minutes followed by 40 cycles at 95°C for 15 seconds and 60°C for 1 minute. All reactions were run in triplicate. After the reactions were complete, the Ct values were determined using fixed threshold settings, and the mean Ct was determined from the triplicate PCRs. The relative expression of mRNAs was determined using the 2^ΔΔCt^ method, and β-actin mRNA served as the internal control. The primer sequences are listed in Supplemental Table 5.

### Protein extraction and Western blotting.

Cells were rinsed with cold PBS (pH 7.4) and then lysed in RIPA buffer (0.5% NP-40, 0.1% sodium deoxycholate, 150 mM NaCl and 50 mM Tris-HCl, pH 7.5) supplemented with a protease and phosphatase inhibitor cocktail (Thermo Fisher Scientific) on ice for 30 minutes. The tissue samples were flash-frozen in liquid nitrogen, ground into powder, and then lysed in RIPA buffer. The cell lysates and tissue homogenates were centrifuged for 10 minutes (12,000*g* at 4°C), the supernatant was collected, and the protein concentration was determined using a Pierce BCA Protein Assay Kit (Thermo Fisher Scientific). Equal amounts of protein (30–60 μg) were resolved via 10%–12.5% SDS-PAGE and then transferred onto a PVDF membrane (MilliporeSigma). The membrane was blocked in Tris-buffered saline Tween-20 (TBST) containing 5% BSA and then incubated with the corresponding primary antibodies overnight at 4°C. After a 1-hour incubation with an HRP-conjugated secondary antibody, the protein level was detected using a luminal reagent. The data were quantified using ImageJ software (NIH), and relative protein expression was normalized to the GAPDH value. The primary antibodies used are listed in Supplemental Table 3.

For analyses of 3 different proteins (CD47, p-STAT3, and STAT3) and 1 internal control (GAPDH) in the same samples, sliced bands from the same blot were used. On the basis of the apparent molecular weights of CD47, p-STAT3, STAT3, and GAPDH (40~70, ~88, ~88, and 37 kDa), the PVDF membrane was cut at 40 kDa and 70 kDa into 3 parts (<40 kDa, 40~70 kDa, and >70 kDa). The 3 parts were then blotted with CD47 (40~70 kDa), p-STAT3 (~88 kDa), and GAPDH (37 kDa) primary antibodies and detected with a secondary antibody. The upper PVDF membrane (>70 kDa) was then treated with antibody removal solution (Beyotime Biotechnology) to remove both primary and secondary antibodies and blotted with a STAT3 (~88 kDa) antibody. For analysis of KRAS (21 kDa), p-STAT3, and STAT3 in the same samples, the PVDF membrane was cut at 35 kDa and 70 kDa into 3 parts (<35 kDa, 35~70 kDa, and > 70 kDa). The 3 parts were then blotted with KRAS (21 kDa), p-STAT3 (~88 kDa), and GAPDH (37 kDa), and the upper PVDF membranes (>70 kDa) were then stripped and blotted with a STAT3 (~88 kDa) antibody. The same experiment was repeated 3 times, and in each biological replicate, the sliced membranes were stripped only once (blotted twice).

### Luciferase reporter assay.

For analysis of the direct binding of miR-34a to CD47, the 3′-UTR of CD47 was inserted into a firefly luciferase reporter plasmid (GenScript). For determination of the binding specificity, sequences that interacted with the miR-34a seed sequence were mutated from ACTGCC, CACTGCC, and ACTGCC to TGACGG, GTGACGG, and TGACGG, respectively, and the mutant CD47 3′-UTR fragment was inserted into the same reporter plasmid. The β-gal plasmid was included as a transfection control. In the luciferase assay, HEK293T cells were cultured in DMEM containing 10% FBS and seeded in 24-well plates. Twenty-four hours after plating, 0.2 μg WT or the mutant luciferase reporter plasmid, 0.1 μg β-gal plasmid, and equal amounts (20 pmol) of the miR-34a mimic or the control mimic (GenePharma) were cotransfected into cells with Lipofectamine 2000 (Invitrogen, Thermo Fisher Scientific) according to the manufacturer’s instructions. Twenty-four hours after transfection, the cells were analyzed using a luciferase assay kit (catalog E4550, Promega) to determine the fluorescence intensity. All experiments were performed in triplicate wells for each condition and repeated 3 times independently.

### In vitro phagocytosis assay.

PBMCs from healthy donors were isolated via density-gradient centrifugation using Ficoll-Hypaque (GE Healthcare). CD14^+^ monocytes were isolated by magnetic column purification on the basis of positive selection with anti-CD14 microbeads (Miltenyi Biotec) with a purity of 96%. Then, 1 × 10^6^ CD14^+^ cells were cultured in RPMI 1640 medium supplemented with 2 mmol/mL glutamine, 100 μg/mL ticarpen, and 10% FBS (complete RPMI) and stimulated with GM-CSF at 25 ng/mL for 7 days to generate macrophages.

The phagocytosis assay was conducted as previously described (19, 35). Briefly, macrophages were plated at a density of 5 × 10^4^ cells per well in a 24-well tissue culture plate in complete DMEM supplemented with GM-CSF overnight before the experiment. H358 cells were pretransfected with the *KRAS^G12C^* plasmid, the miR-34a mimic, or the *CD47* plasmid and their corresponding negative controls for 48 hours and then stained with 2.5 μM CFSE at 37°C for 10 minutes. Macrophages were incubated in serum-free medium for 2 hours before addition of 2 × 10^5^ CFSE-labeled H358 cells. After coculturing for 2 hours at 37°C, the cells were harvested, the macrophages were stained with APC-labeled anti-F4/80 antibody (BD Biosciences), and flow cytometry (FACScalibur, BD Biosciences) was performed to detect CFSE^+^F4/80^+^ cells. A total of 10,000 cells in each sample were analyzed. Phagocytosis was calculated as the percentage of CFSE^+^F4/80^+^ cells (Q2) among CFSE^+^ cells (Q1 + Q2): phagocytosis (percentage) = [Q2/(Q1 + Q2)] × 100%.

For direct visualization of the phagocytosed H358 cells by macrophages, a phagocytosis assay was performed by fluorescence microscopy (36). Briefly, a GFP-encoding lentivirus was prepared from the pCDH-CMV construct using standard techniques and transfected into H358 cells to generate GFP^+^ cells. Macrophages were plated at a density of 5 × 10^4^ cells per well in a 24-well tissue culture plate. GFP^+^ H358 cells were pretransfected with the *KRAS^G12C^* plasmid, the miR-34a mimic, or the *CD47* plasmid and their corresponding negative controls for 48 hours. Macrophages were incubated in serum-free medium for 2 hours. Then, 2 × 10^4^ GFP^+^ H358 cells were added to the macrophage-containing wells and incubated for 2 hours at 37°C. Macrophages were repeatedly washed and subsequently examined by fluorescence microscopy (Leica DMI6000B). Macrophages that were GFP^+^ represent macrophages containing phagocytosed H358 cells. The phagocytic index was calculated as the number of phagocytosed GFP^+^ cells per 100 macrophages.

### IHC analysis in tissue microarray.

Commercial tissue microarray chips containing 157 pairs of lung adenocarcinoma samples and normal adjacent tissue (NAT) samples were purchased from Shanghai Outdo Biotech. Each sample dot with a diameter of 1.5 mm and a thickness of 4 μm was prepared according to a standard method. All patients had been pathologically diagnosed with adenocarcinoma after operation, and follow-up data (range 0–120 months) were available. Informed consent was obtained for all patients. The IHC analysis was performed as described previously (37). Briefly, the tissue sections were blocked with goat serum and then incubated with anti-CD47 (1:100, Abcam, ab175388), anti–p-STAT3 (1:100, 9145S, Cell Signaling Technology), or anti–p-AKT (1:100, 4066S, Cell Signaling Technology) antibodies overnight at 4°C. The sections were stained with 3,3-diaminobenzidine and counterstained with hematoxylin after being incubated with a secondary antibody. All IHC sample dots were assessed by 2 independent pathologists blinded to both the sample origins and the patient outcomes. Both staining intensity and positive percentage were used to assess the expression of CD47, p-STAT3, and p-AKT in the lung cancer tissues: IHC staining was scored according to the extent of cell staining (≤10% positive cells = 0; 11%–50% positive cells = 2; 51%–80% positive cells = 3; >80% positive cells = 4) and the staining intensity (no staining = 0; slight staining = 1; moderate staining = 2; strong staining = 3). Scores for the percentage of positive cells and the staining intensity were added. The CD47, p-STAT3, and p-AKT expression levels in the lung adenocarcinoma tissues were considered medium expression when the score of each protein was in the range of an average score ± 20% in all samples; high expression was considered higher than medium expression; and low expression was considered lower than medium expression. Patient information related to the tissue microarray is shown in Supplemental Table 2.

In addition, a tissue microarray containing 12 pairs of *KRAS^MUT^* lung adenocarcinoma and normal adjacent tissue samples and 28 pairs of *KRAS^WT^* lung adenocarcinoma and normal adjacent tissue samples was obtained from the Jiangsu Biobank of Clinical Resources. All patients had been pathologically diagnosed with adenocarcinoma after their operation, and informed consent was obtained from all patients. IHC analysis in the tissue microarray was performed with anti-CD47 and anti-p-STAT3 antibodies as described above. Patient information related to the tissue microarray is shown in Supplemental Table 2.

### Statistics.

All statistical tests were performed using the open-source statistics package R or GraphPad Prism 8 (GraphPad Software). All data are presented as the mean ± SEM. Differences were considered statistically significant at a *P* value of less than 0.05. Normality and equal variances between group samples were assessed using the Shapiro-Wilk test and Brown–Forsythe tests, respectively. When normality and equal variance were achieved between sample groups, 1-way ANOVA (followed by Bonferroni’s multiple-comparison test), 2-way ANOVA (followed by Bonferroni’s multiple-comparison test), or *t* tests were used. Where normality or equal variance of samples failed, Kruskal–Wallis 1-way ANOVA (followed by Dunn’s correction) or Mann-Whitney *U* tests were performed.

### Study approval.

All patient samples were obtained from the Jiangsu Biobank of Clinical Resources (located at Jiangsu Cancer Hospital, Nanjing, China). These samples were collected from patients with lung adenocarcinoma who underwent surgery at the Jiangsu Cancer Hospital. Informed consent was obtained from each patient, and the collection of tissue specimens was approved by the internal review and ethics boards of Jiangsu Cancer Hospital. All animal care and handling procedures were performed in accordance with the NIH’s *Guide for the Care and Use of Laboratory Animals* (National Academies Press, 2011) and were approved by the ethics committee of Nanjing University (Nanjing, China).

## Author contributions

XC, CY, and R Yin conceptualized the study. HH, RC, YW, XW, JW, YK, SZ, ZZ, HZ, R Yu, GL, QW, and XZ designed the study methodology. HH, RC, YW, XW, JW, YK, SZ, ZZ, HZ, RY, GL, QW, and XZ conducted experiments. HH, RC, and YW performed visualization studies. XC, CY, and RY acquired funding. XC, CY, and RY were responsible for project administration. XC, CY, and R Yin supervised the study. HH and RC wrote the original draft of the manuscript. XC, CY, and CYZ reviewed and edited the manuscript. The order of the co–first authors was determined according to the time spent on this project.

## Supplementary Material

Supplemental data

## Version 1 11/22/2022

In-Press Preview

## Version 2 01/17/2023

Electronic publication
