# Supplementary material for: Oncogenic KRAS signaling drives evasion of innate immune surveillance in lung adenocarcinoma by activating CD47
Source: J Clin Invest. 2023 Jan 17;133(2):e153470. doi: 10.1172/JCI153470 (PMC9843062; doi:10.1172/JCI153470)
Supplement: Supplemental data [file jci-133-153470-s147.pdf]

Supplementary materials for

Oncogenic KRAS signaling drives evasion of innate immune surveillance in lung adenocarcinoma by activating CD47

(Huanhuan Hu *et al.*)

This file includes:

Supplementary Figure 1 to Supplementary Figure 16

Supplementary Table 1 to Supplementary Table 5

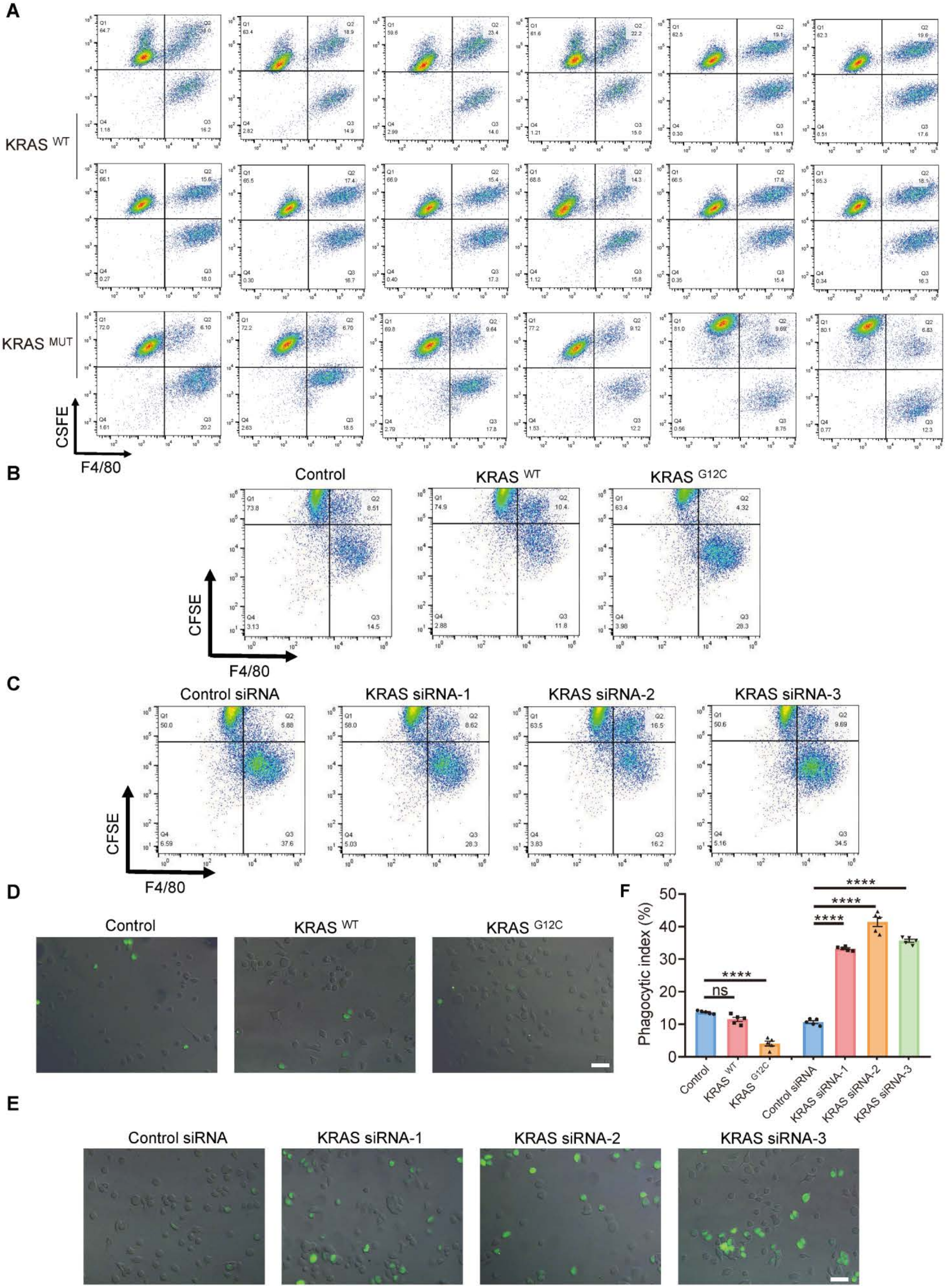
A
KRAS WT
KRAS MUT
CSFE
F4/80
B
Control
KRAS WT
KRAS G12C
CFSE
F4/80
C
Control siRNA
KRAS siRNA-1
KRAS siRNA-2
KRAS siRNA-3
CFSE
F4/80
D
Control
KRAS WT
KRAS G12C
F
Phagocytic index (%)
ns
****
Control
KRAS WT
KRAS G12C
Control siRNA
KRAS siRNA-1
KRAS siRNA-2
KRAS siRNA-3
E
Control siRNA
KRAS siRNA-1
KRAS siRNA-2
KRAS siRNA-3

Supplementary Figure 1. Oncogenic KRAS mutations render lung cancer cells insensitive to macrophage phagocytosis. (**A**) Tumor cells from $KRAS^{MUT}$ lung adenocarcinoma patients were more resistant to macrophage phagocytosis than that from $KRAS^{WT}$ patients. Cancer cells were isolated from 12 $KRAS^{WT}$ and 6 $KRAS^{MUT}$ patients, labeled with the fluorescent dye CFSE, incubated with human peripheral blood monocyte-derived macrophages, stained with F4/80, and analyzed by flow cytometry. Phagocytosis rate was calculated as the percentage of $CFSE^{+}F4/80^{+}$ cells among the $CFSE^{+}$ cells. FACS results for all 18 patients are shown. (**B-C**) Effect of KRAS manipulation on macrophage phagocytosis of $KRAS^{G12C}$ H358 cells. H358 cells were transfected with plasmids expressing $KRAS^{WT}$ or $KRAS^{G12C}$ or with three *KRAS* siRNAs. After 48 h, the cells were labeled with CFSE and subjected to in vitro phagocytosis assay. Representative FACS result for each group is shown. (**D-F**) Effect of KRAS manipulation on macrophage phagocytosis of $KRAS^{G12C}$ H358 cells. Fluorescence microscopy of the macrophage phagocytosis of the eGFP-labeled H358 cells transfected with plasmids expressing $KRAS^{WT}$ or $KRAS^{G12C}$ or with three *KRAS* siRNA for 48 h before coculture with human peripheral blood monocyte-derived macrophages. Representative fluorescence images (**D, E**; scale bar, 50 μm) and phagocytic index (**F**; n = 5 per group) are shown. Data are shown as the mean ± SEM. ****$P < 0.0001$, by one-way ANOVA (**F**).

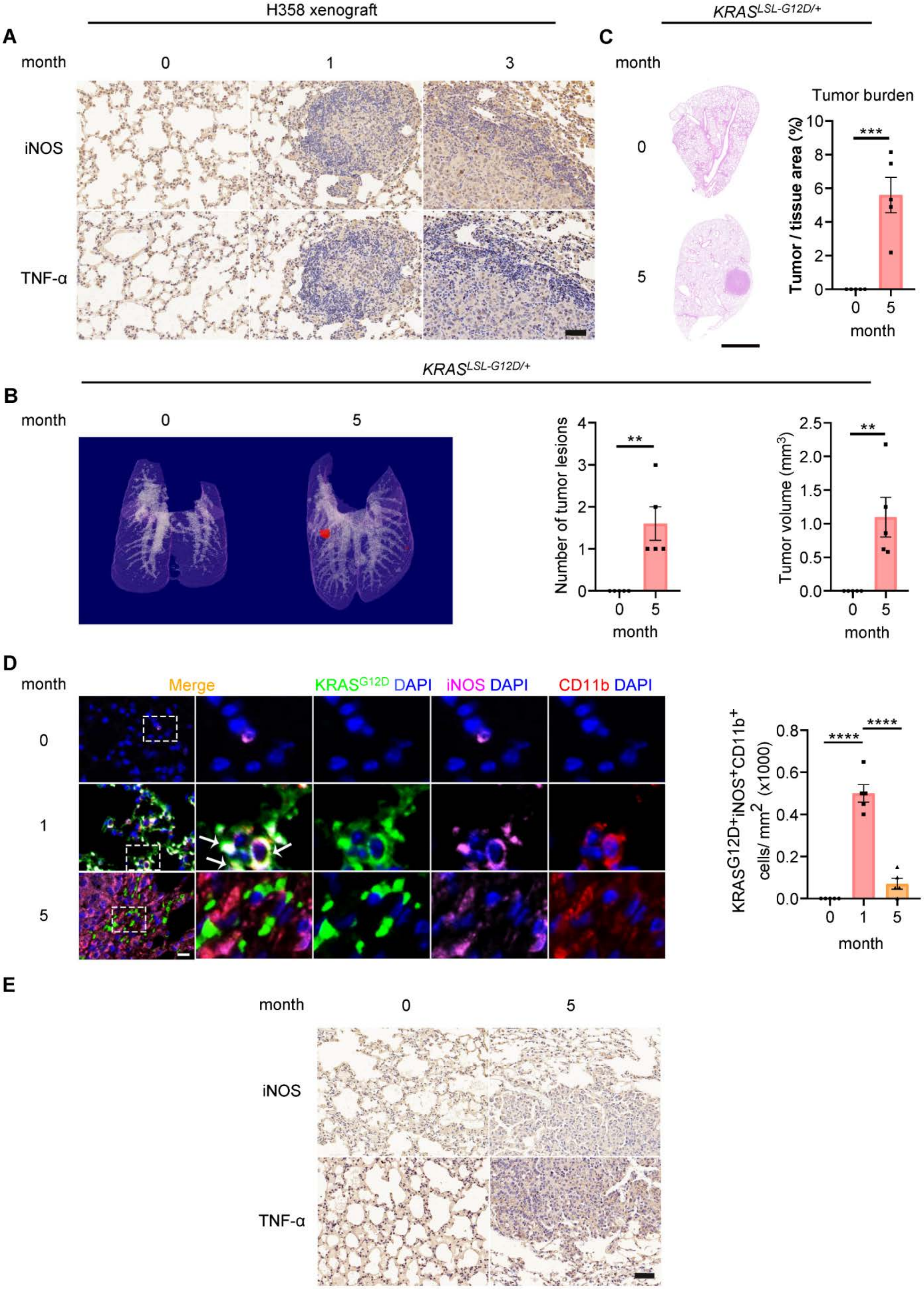
H358 xenograft
A
month
0
1
3
iNOS
TNF-α
KRAS LSL-G12D/+
C
month
0
5
Tumor burden
Tumor / tissue area (%)
month
***
B
KRAS LSL-G12D/+
month
0
5
Number of tumor lesions
Tumor volume (mm3)
month
**
D
month
Merge
KRASG12D DAPI
iNOS DAPI
CD11b DAPI
0
1
5
KRASG12D+iNOS+CD11b+ cells/ mm2 (x1000)
month
****
E
month
0
5
iNOS
TNF-α

Supplementary Figure 2. Macrophage phagocytosis of tumor cells is inhibited in the H358 lung colonization model and *Kras*$^{LSL\text{-}G12D/+}$ mice. (**A**) IHC staining of the M1 macrophage markers iNOS and TNF-α in the lung sections derived from the H358 lung colonization model at different time points. Scale bar, 20 μm. (**B-E**) Monitoring of tumor growth and macrophage infiltration in *Kras*$^{LSL\text{-}G12D/+}$ mice. *Kras*$^{LSL\text{-}G12D/+}$ mice were intratracheally administered Adeno-Cre to trigger pulmonary adenocarcinoma formation. (**B**) Tumor growth was monitored by non-invasive micro-CT scans at different time points post-administration. Left panel: representative 3-D reconstructions of the mouse lungs. Right panel: quantitative analysis of the tumor numbers and volumes (n = 5 per group). (**C**) Tumor growth was also evaluated by H&E-staining of lung sections. Left panel: representative H&E images. Scale bar, 2 mm. Right panel: quantification of tumor burden (n = 5 per group). (**D**) Macrophage infiltration was assessed by immunofluorescence staining of CD11b (red), iNOS (purple), KRAS^{G12D} (green) and DAPI (blue) in the mouse lung tumor tissue. Left panel: representative images. Scale bar, 50 μm. Right panel: quantification results (n = 5 mice per group). (**E**) The presence of M1 macrophages in lung tumor tissue was assessed by IHC staining of the M1 macrophage markers iNOS and TNF-α in the lung sections from *Kras*$^{LSL\text{-}G12D/+}$ mice. Scale bar, 20 μm. Data are shown as the mean ± SEM. **P < 0.01, ***P < 0.001 and ****P < 0.0001, by unpaired t test (**B-C**) or one-way ANOVA (**D**).

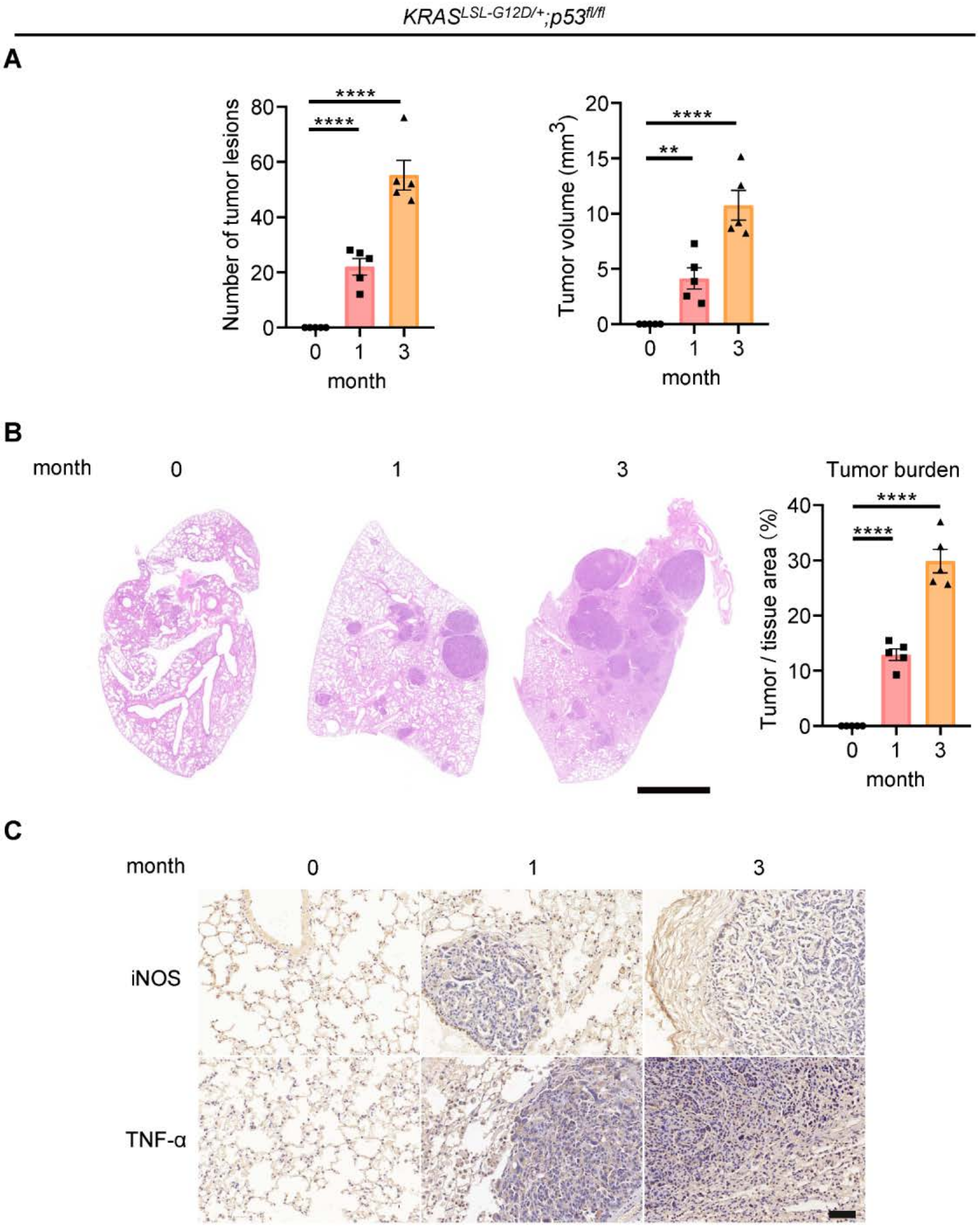

Supplementary Figure 3. Macrophage phagocytosis of tumor cells is inhibited in *Kras$^{LSL\text{-}G12D/+}$;p53$^{fl/fl}$* mice. *Kras$^{LSL\text{-}G12D/+}$;p53$^{fl/fl}$* mice were intratracheally administered Adeno-Cre to trigger pulmonary adenocarcinoma formation. (**A**) Tumor growth was monitored by non-invasive micro-CT scans at different time points post-administration. Quantitative analysis of the tumor numbers and volumes (n = 5 per group) is shown. (**B**) Tumor growth was evaluated by H&E-staining of lung sections. Left panel: representative H&E images. Scale bar, 2 mm. Right panel: quantification of tumor burden (n = 5 per group). (**C**) The presence of M1 macrophages in lung tumor tissue was assessed by IHC staining of the M1 macrophage markers iNOS and TNF-α in the lung sections from *Kras$^{LSL\text{-}G12D/+}$;p53$^{fl/fl}$* mice. Scale bar, 20 μm. Data are shown as the mean ± SEM. **P < 0.01 and ****P < 0.0001, by one-way ANOVA (**A-B**).

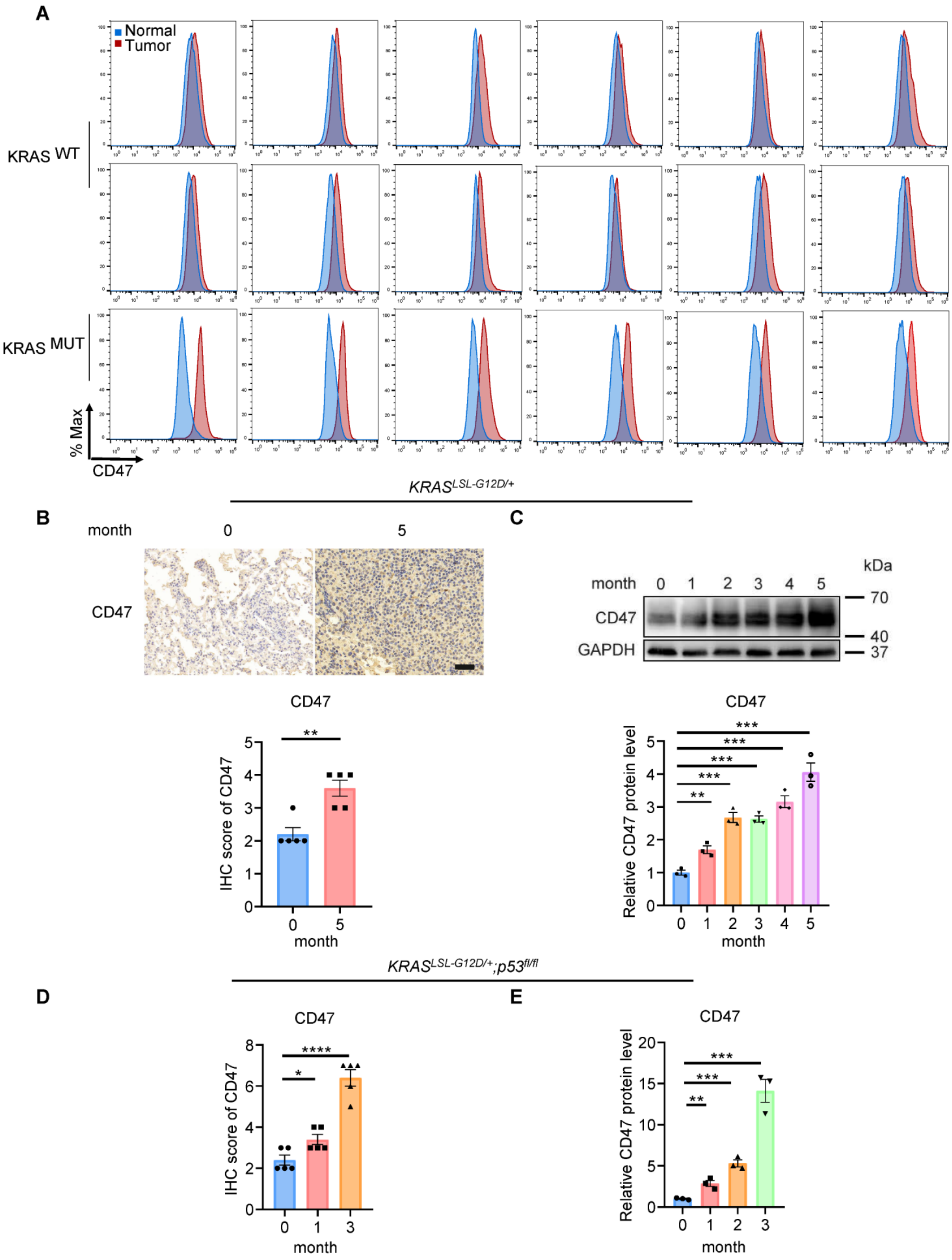
A
Normal
Tumor
KRAS WT
KRAS MUT
% Max
CD47
KRAS$^{LSL-G12D/+}$
B
month
0
5
CD47
C
kDa
month 0 1 2 3 4 5
CD47
GAPDH
70
40
37
CD47
IHC score of CD47
month
Relative CD47 protein level
KRAS$^{LSL-G12D/+}$;p53$^{fl/fl}$
D
E

Supplementary Figure 4. KRAS mutations drives CD47 expression in lung adenocarcinoma patients and mouse models of lung cancer. (**A**) FACS analysis of surface expression of CD47 on tumor cells isolated from 12 *KRAS*^{WT} and 6 *KRAS*^{MUT} lung adenocarcinoma patients. (**B**) IHC analysis of the CD47 protein levels in the lung tumors of *Kras*$^{LSL\text{-}G12D/+}$ mice. Representative images (scale bar, 20 μm) and quantification results (n = 5 per group) are shown. (**C**) Immunoblotting analysis of CD47 protein levels in the lung tumors of *Kras*$^{LSL\text{-}G12D/+}$ mice. Representative images and quantification results (n = 3 per group) are shown. (**D**) IHC analysis of the CD47 protein levels in the lung tumors from *Kras*$^{LSL\text{-}G12D/+}$*;p53*$^{fl/fl}$ mice. Quantification results (n = 5 per group) are shown. (**E**) Western blot analysis of CD47 protein levels in the lung tumors from *Kras*$^{LSL\text{-}G12D/+}$*;p53*$^{fl/fl}$ mice. Quantification results (n = 3 per group) are shown. Data are shown as the mean ± SEM. *P < 0.05, **P < 0.01, ***P < 0.001 and ****P < 0.0001, by unpaired t test (**B**) or one-way ANOVA (**C-E**).

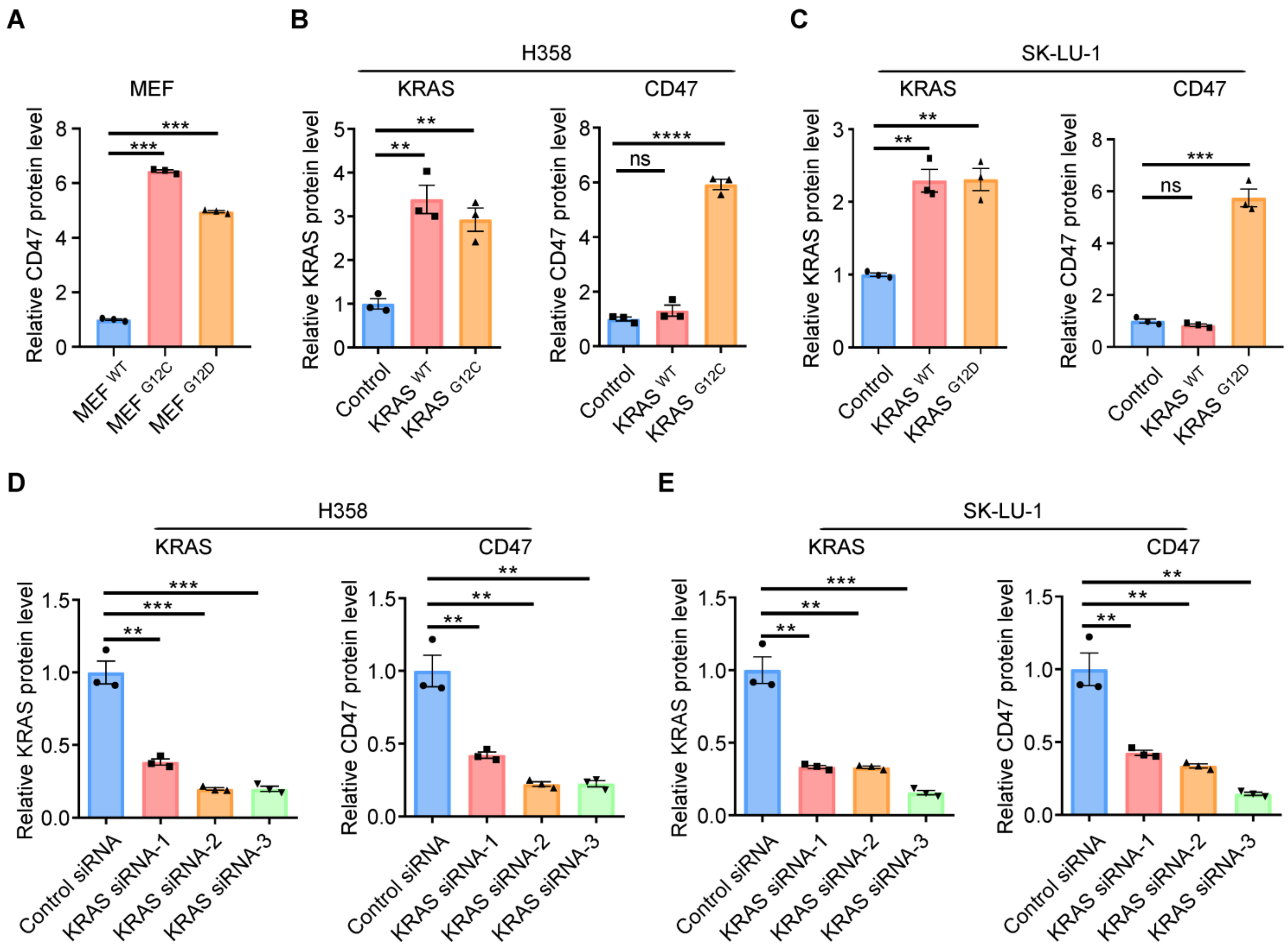

Supplementary Figure 5. KRAS mutations drive CD47 protein expression in vitro. (**A**) Immunoblotting analysis of the CD47 protein levels in MEFs overexpressing $KRAS^{WT}$, $KRAS^{G12C}$ or $KRAS^{G12D}$. Quantification results (n = 3 per group) are shown. (**B-C**) Immunoblotting analysis of KRAS and CD47 protein levels in H358 (**B**) and SK-LU-1 (**C**) cells transfected with plasmids expressing $KRAS^{WT}$ or $KRAS^{MUT}$. Quantification results (n = 3 per group) are shown. (**D-E**) Immunoblotting analysis of KRAS and CD47 protein levels in H358 (**D**) and SK-LU-1 (**E**) cells transfected with three *KRAS* siRNAs. Quantification results (n = 3 per group) are shown. Data are shown as the mean ± SEM. $^{**}P < 0.01$and $^{***}P < 0.001$, by one-way ANOVA (**A-E**).

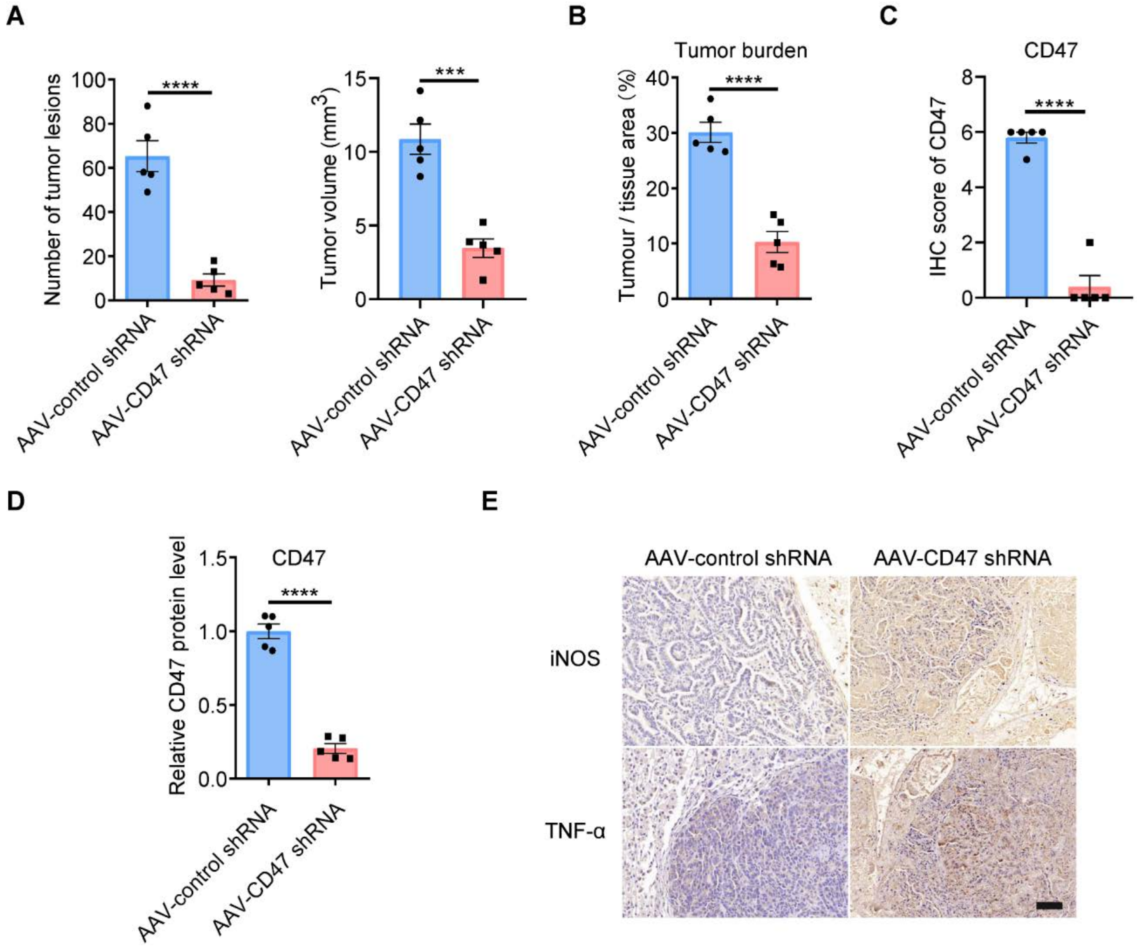

Supplementary Figure 6. Silencing of CD47 expression by shRNA induces tumor regression in the *Kras*$^{LSL\text{-}G12D/+}$*;p53*$^{fl/fl}$ mice. The *Kras*$^{LSL\text{-}G12D/+}$*;p53*$^{fl/fl}$ mice were intratracheally administered Adeno-Cre along with AAV-control shRNA or AAV-*Cd47* shRNA. The mice were then monitored to evaluate tumor growth and CD47 expression. (**A**) Quantification of the tumor numbers and volumes at 3 months post-administration from the micro-CT images (n = 5 per group). (**B**) Quantification of the tumor area from H&E-stained lung sections (n = 5 per group). (**C**) IHC analysis of the CD47 protein levels in the lung tumors. Quantitative results of the IHC scores are shown (n = 5 mice per group). (**D**) Immunoblotting analysis of the CD47 protein levels in the lung tumors. Quantitative results are shown (n = 5 mice per group). (**E**) IHC staining of the M1 macrophage markers iNOS and TNF-α in the lung sections derived from the *Kras*$^{LSL\text{-}G12D/+}$*;p53*$^{fl/fl}$ mice treated with AAV-*Cd47* shRNA. Scale bar, 20 μm. Data are shown as the mean ± SEM. ***P < 0.001 and ****P < 0.0001, by unpaired t test (**A-D**).

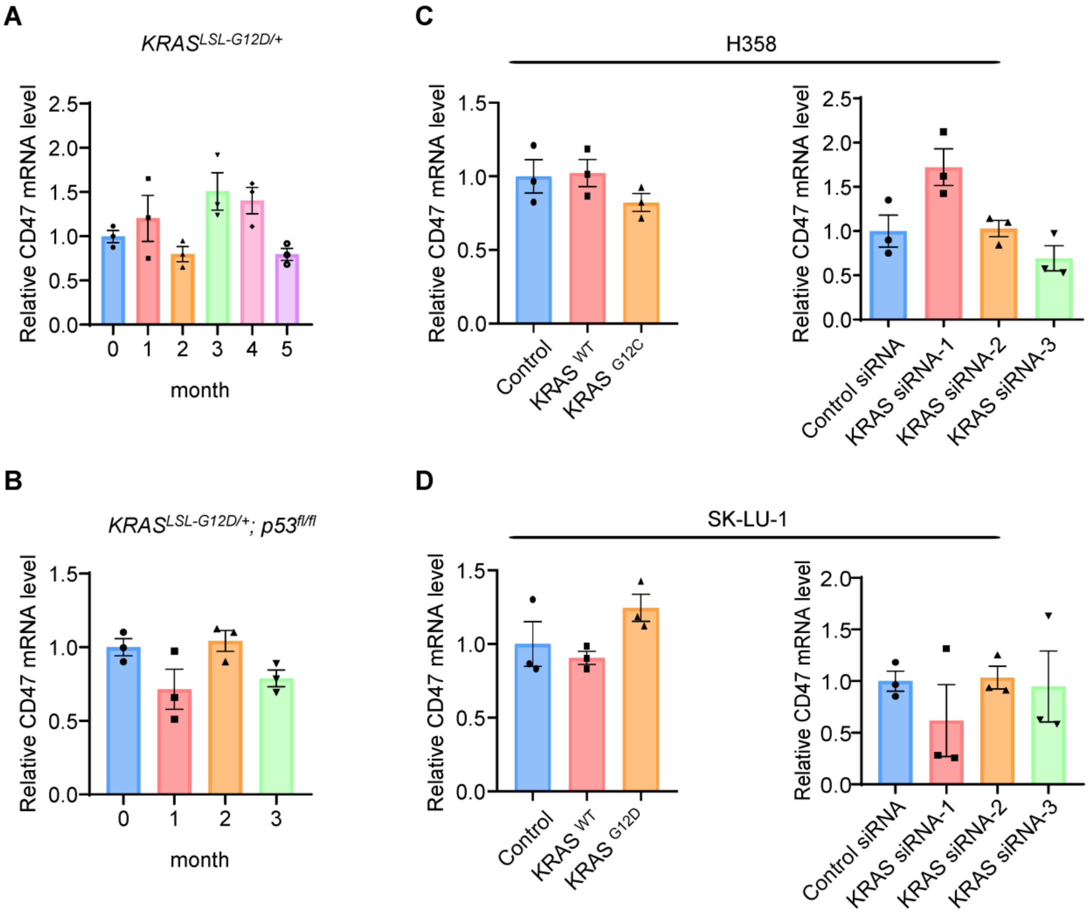

Supplementary Figure 7. Effect of *KRAS* mutation status and expression on CD47 mRNA levels in vitro and in vivo. (**A-B**) Quantitative RT-PCR analysis of the relative *Cd47* mRNA levels in the whole lung extracts from the $Kras^{LSL\text{-}G12D/+}$ and $Kras^{LSL\text{-}G12D/+}$;$p53^{fl/fl}$ mice at different time points (n = 3 per group). (**C-D**) Quantitative RT-PCR analysis of the relative *CD47* mRNA levels in the H358 (**C**) and SK-LU-1 (**D**) cells that were transfected with the plasmids expressing $KRAS^{WT}$ or $KRAS^{MUT}$ or with three *KRAS* siRNAs (n = 3 per group). Data are shown as the mean ± SEM. The differences among groups are not significant by one-way ANOVA analysis (**A-D**).

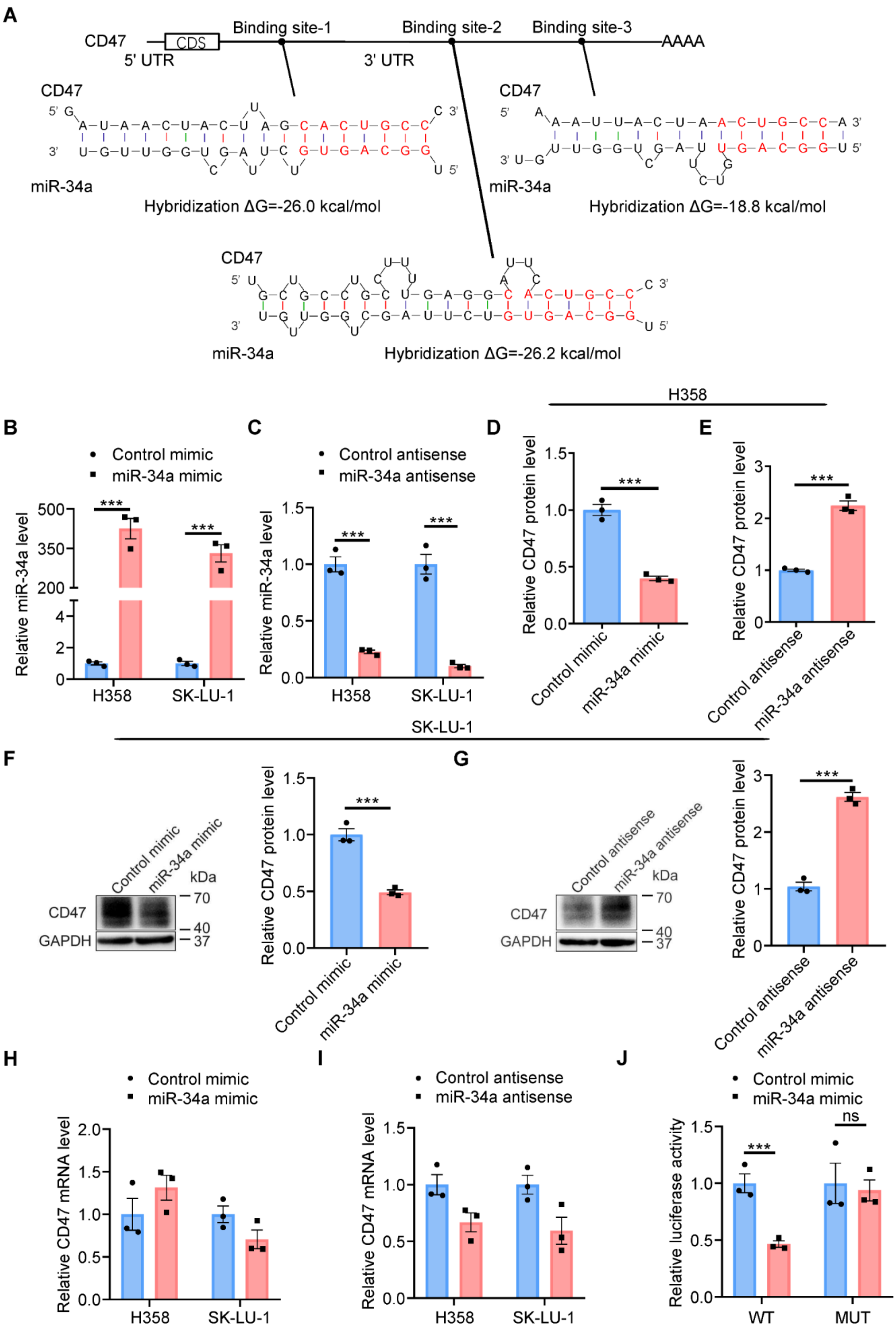
A
CD47
CDS
5' UTR
Binding site-1
3' UTR
Binding site-2
Binding site-3
AAAA
miR-34a
Hybridization ΔG=-26.0 kcal/mol
Hybridization ΔG=-18.8 kcal/mol
Hybridization ΔG=-26.2 kcal/mol
H358
B
Control mimic
miR-34a mimic
Relative miR-34a level
C
Control antisense
miR-34a antisense
D
Relative CD47 protein level
E
SK-LU-1
F
CD47
GAPDH
kDa
G
H
Relative CD47 mRNA level
I
J
Relative luciferase activity
WT
MUT
ns

Supplementary Figure 8. Post-transcriptional regulation of CD47 by miR-34a. (**A**) Schematic description of the predicted duplexes formed by miR-34a and the 3'-UTR of *CD47* mRNA. The predicted free energy values of the hybrids are indicated, which were well within the range of genuine miRNA-target pairs. (**B**) Quantitative RT-PCR analysis of the relative expression levels of miR-34a in H358 and SK-LU-1 cells transfected with control or miR-34a mimic (n = 3 per group). (**C**) Quantitative RT-PCR analysis of the relative expression levels of miR-34a in H358 and SK-LU-1 cells transfected with control or miR-34a antisense (n = 3 per group). (**D**) Immunoblotting analysis of CD47 protein levels in H358 cells transfected with control or miR-34a mimic. Quantification results (n = 3 per group) are shown. (**E**) Immunoblotting analysis of CD47 protein levels in H358 cells transfected with control or miR-34a antisense. Quantification results (n = 3 per group) are shown. (**F**) Immunoblotting analysis of CD47 protein levels in SK-LU-1 cells transfected with control or miR-34a mimic. Left panel: representative blots. Right panel: quantification results (n = 3 per group). (**G**) Immunoblotting analysis of CD47 protein levels in SK-LU-1 cells transfected with control or miR-34a antisense. Left panel: representative blots. Right panel: quantification results (n = 3 per group). (**H**) Quantitative RT-PCR analysis of the relative *CD47* mRNA levels in H358 and SK-LU-1 cells transfected with control or miR-34a mimic (n = 3 per group). (**I**) Quantitative RT-PCR analysis of the relative *CD47* mRNA levels in H358 and SK-LU-1 cells transfected with the control or miR-34a antisense (n = 3 per group). (**J**) Firefly luciferase reporters containing wild-type or mutant miR-34a binding sites in the CD47 3'-UTR were co-transfected into HEK293T cells together with control or miR-34a mimic. The reduction in luciferase activity only in the wild-type reporters indicates direct binding of miR-34a to the presumed sites in the 3'-UTR of CD47 (n = 3 per group). Data are shown as the mean ± SEM. ***$P < 0.001$, by unpaired t test (**A-J**).

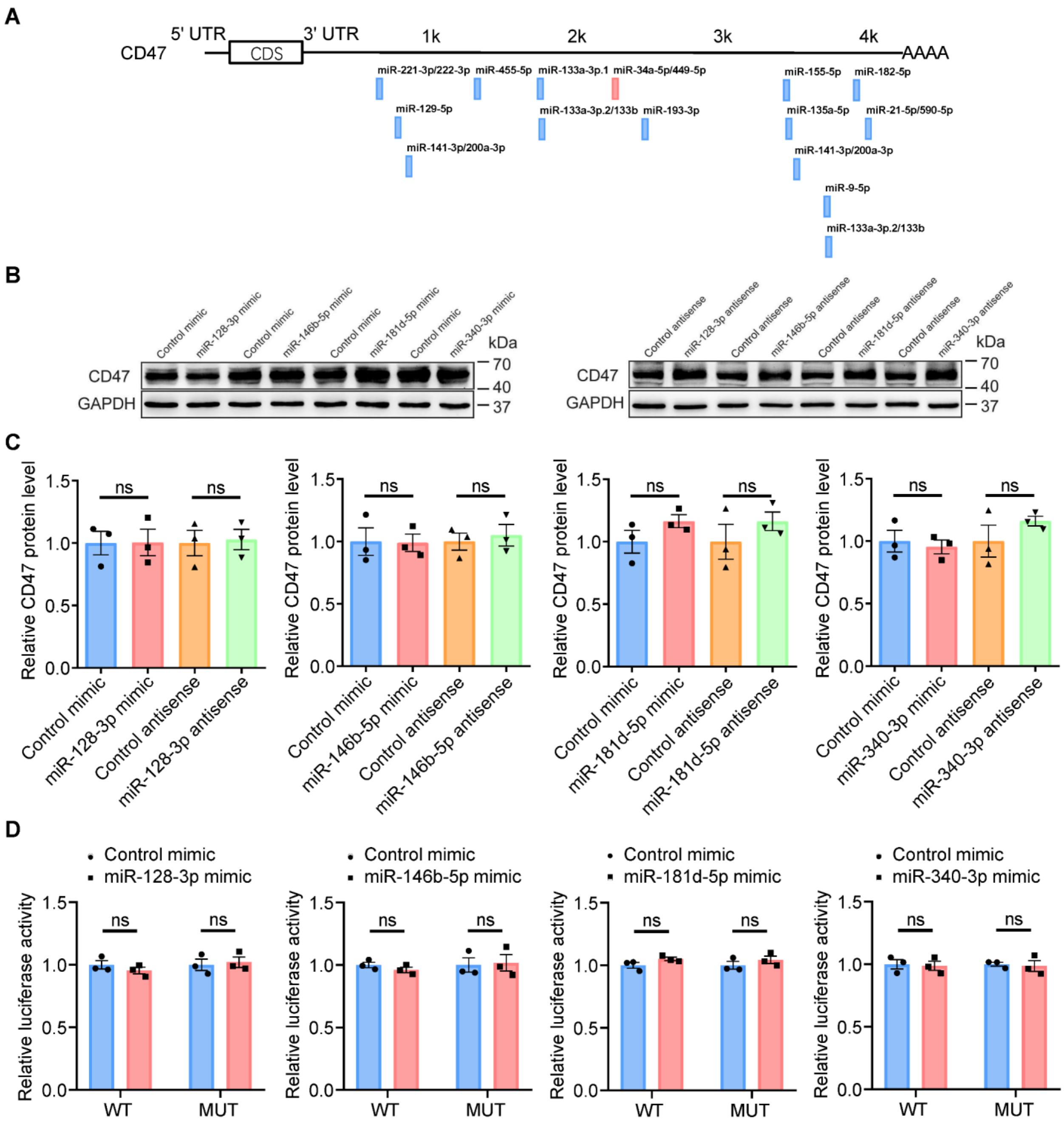

Supplementary Figure 9. Lack of post-transcriptional regulation of CD47 by the other four miRNAs that were downregulated in tumor tissues. (**A**) Schematic description of the predicted miRNAs binding to *CD47* mRNA. (**B**) Immunoblotting analysis of CD47 protein levels in H358 cells with miRNA manipulation. Left panel: cells were transfected with control or miRNA mimic; right panel: cells were transfected with control or miRNA antisense. (**C**) Quantification results from (**B**) are shown (n = 3 per group). (**D**) Firefly luciferase reporters containing wild-type or mutant miRNAs binding sites in the CD47 3'-UTR were co-transfected into HEK293T cells together with the control or miRNA mimics. No reduction in luciferase activity was observed indicating lack of binding of these miRNAs to the 3'-UTR of CD47 (n = 3 per group).

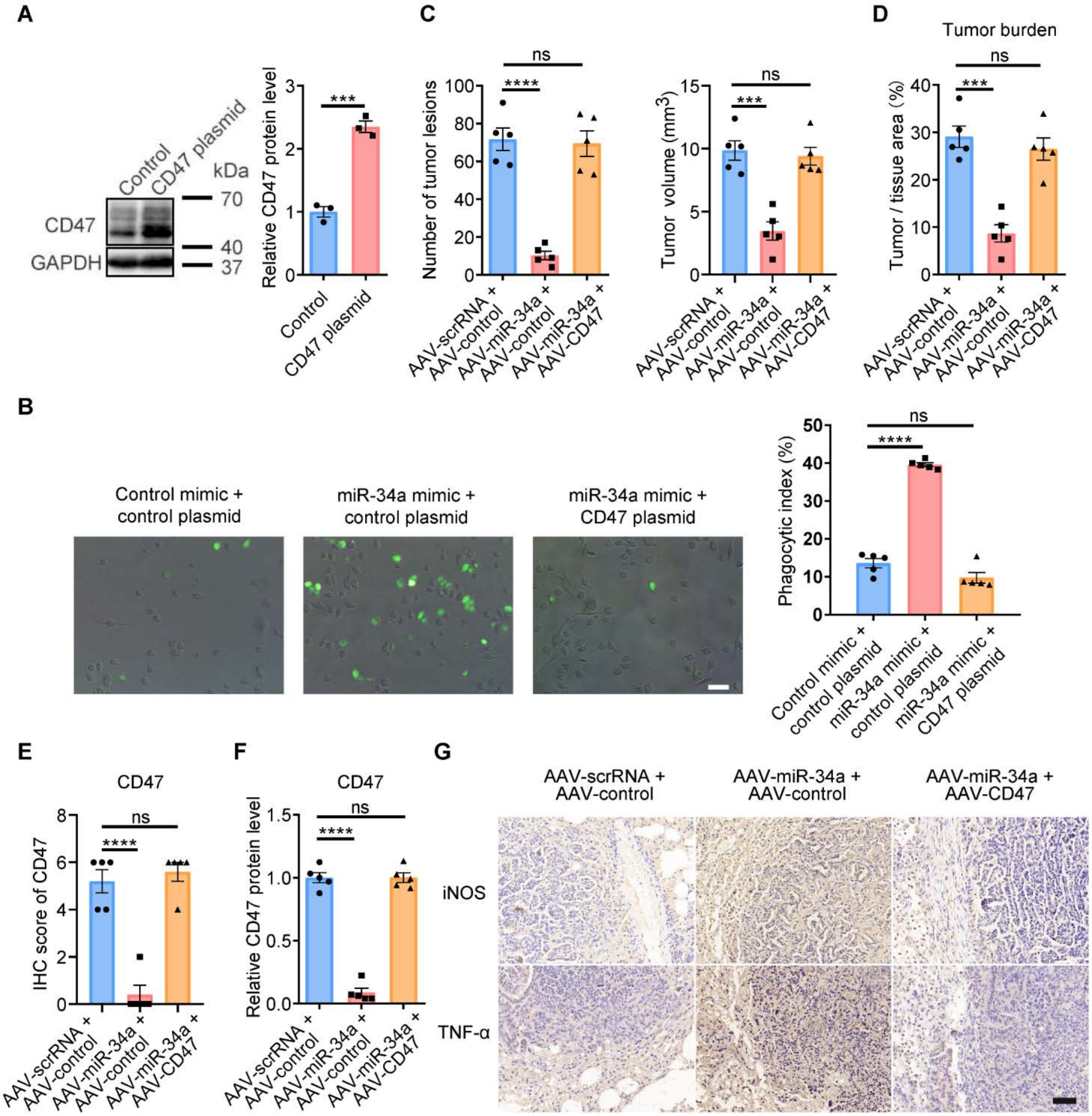

Supplementary Figure 10. MiR-34a restores the phagocytic function of macrophages by negatively regulating CD47 activity in vitro and in vivo. (**A**) Efficient induction of CD47 protein expression in H358 lung cancer cells by a plasmid expressing the CD47 coding sequence (CD47 plasmid). Left panel: representative blots. Right panel: quantification results (n = 3). (**B**) Fluorescence microscopy of the macrophage phagocytosis of the eGFP-labeled H358 cells that were co-transfected with the control mimic plus the control plasmid, the miR-34a mimic plus the control plasmid or the miR-34a mimic plus the CD47 plasmid for 48 h before coculture with human peripheral blood monocyte-derived macrophages. Left panel: representative fluorescence images. Scale bar, 50 μm. Right panel: phagocytic index (n = 5). (**C-G**) MiR-34a restricts CD47 activity and induces tumor regression in the *Kras*$^{LSL-G12D/+}$*;p53*$^{fl/fl}$ mouse model of lung cancer. The *Kras*$^{LSL-G12D/+}$*;p53*$^{fl/fl}$ mice were

intratracheally administered Adeno-Cre along with various combinations of AAV-scrRNA, AAV-control, AAV-miR-34a or AAV-*Cd47*. The mice were then monitored to evaluate tumor growth, CD47 expression and macrophage phagocytosis. (**C**) Quantification of the tumor number and volume from micro-CT images (n = 5). (**D**) Quantification of the tumor burden from H&E staining (n = 5). (**E**) IHC staining of CD47 in the lung adenocarcinoma sections. The quantification of IHC scores is shown (n = 5). (**F**) Immunoblotting analysis of CD47 protein levels in the lung adenocarcinomas. The quantitative analysis is shown (n = 5). (**G**) IHC staining of the M1 macrophage markers iNOS and TNF-α in the lung sections derived from the $Kras^{LSL\text{-}G12D/+};p53^{fl/fl}$ mice treated with AAV-miR-34a alone or together with AAV-Cd47. Scale bar, 20 μm. Data are shown as mean ± SEM. ***P < 0.001 and ****P < 0.0001, by unpaired t test (**A**) or one-way ANOVA (**B-F**).

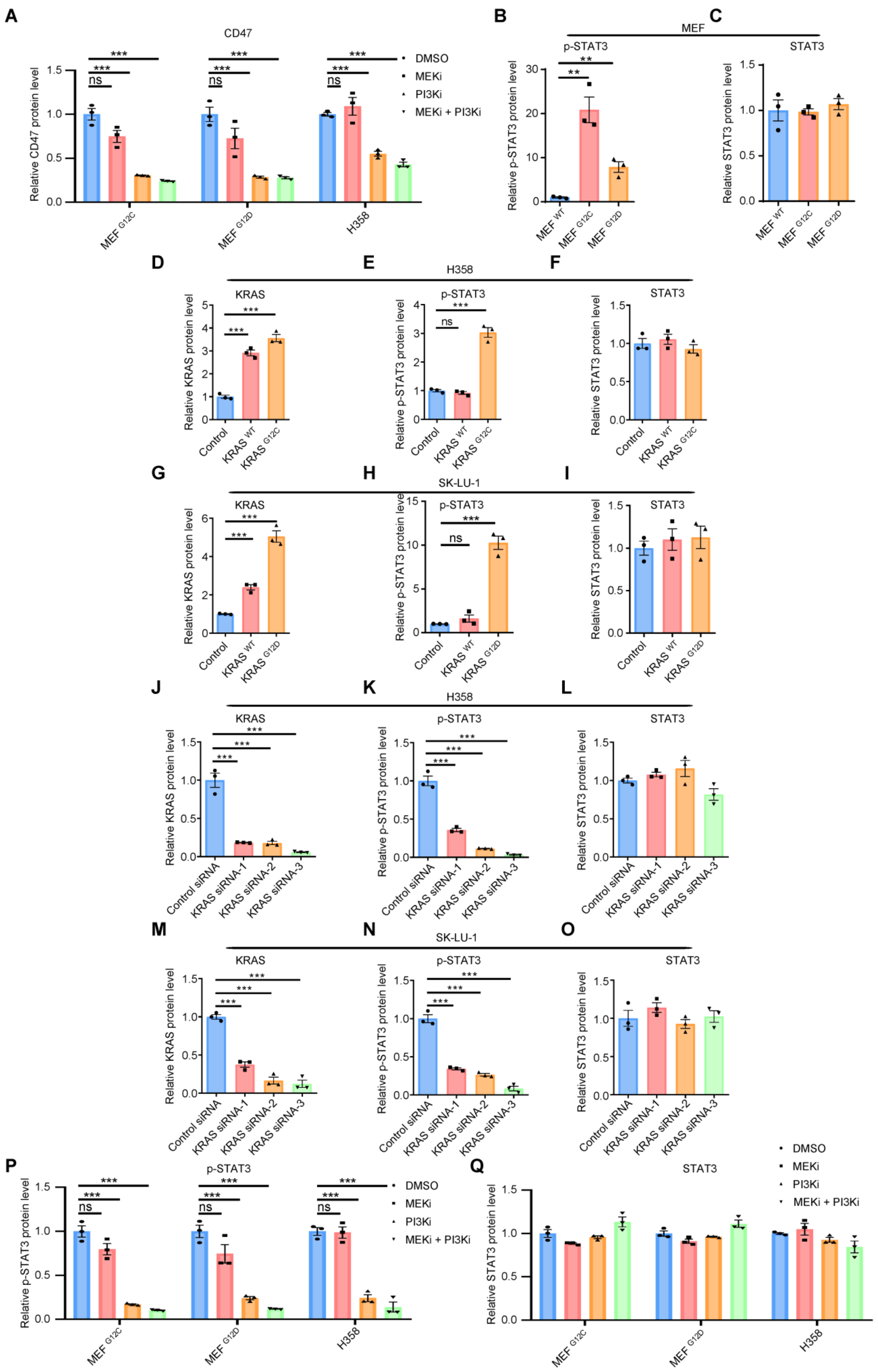
A
CD47
Relative CD47 protein level
DMSO
MEKi
PI3Ki
MEKi + PI3Ki
MEF G12C
MEF G12D
H358
B
C
MEF
p-STAT3
STAT3
Relative p-STAT3 protein level
Relative STAT3 protein level
MEF WT
D
E
F
H358
KRAS
Relative KRAS protein level
Control
KRAS WT
KRAS G12C
G
H
I
SK-LU-1
KRAS G12D
J
K
L
Control siRNA
KRAS siRNA-1
KRAS siRNA-2
KRAS siRNA-3
M
N
O
P
Q

Supplementary Figure 11. *KRAS* mutations lead to the phosphorylation of STAT3, suppression of miR-34a expression and activation of CD47 expression in lung cancer cells. (**A**) Immunoblotting analysis of the expression levels of CD47 in the MEFs and H358 cells treated with MEK inhibitor, PI3K inhibitor or both. Quantification results (n = 3 per group) are shown. (**B-C**) Immunoblotting analysis of the expression levels of p-STAT3 and total STAT3 in MEFs. Quantification results (n = 3 per group) are shown. (**D-F**) Immunoblotting analysis of the expression levels of KRAS, p-STAT3 and total STAT3 in H358 cells overexpressing $KRAS^{WT}$ or $KRAS^{G12C}$. Quantification results (n = 3 per group) are shown. (**G-I**) Immunoblotting analysis of the expression levels of KRAS, p-STAT3 and total STAT3 in SK-LU-1 cells overexpressing $KRAS^{WT}$ or $KRAS^{G12D}$. Quantification results (n = 3 per group) are shown. (**J-L**) Immunoblotting analysis of the expression levels of KRAS, p-STAT3 and total STAT3 in H358 cells transfected with three *KRAS* siRNAs. Quantification results (n = 3 per group) are shown. (**M-O**) Immunoblotting analysis of the expression levels of KRAS, p-STAT3 and total STAT3 in SK-LU-1 cells transfected with three *KRAS* siRNAs. Quantification results (n = 3 per group) are shown. (**P-Q**) Immunoblotting analysis of the expression levels of p-STAT3 and total STAT3 in MEFs and H358 cells treated with MEK inhibitor, PI3K inhibitor or both. Quantification results (n = 3 per group) are shown. Data are shown as the mean ± SEM. **P < 0.01 and ***P < 0.001, by one-way ANOVA (**A-Q**).

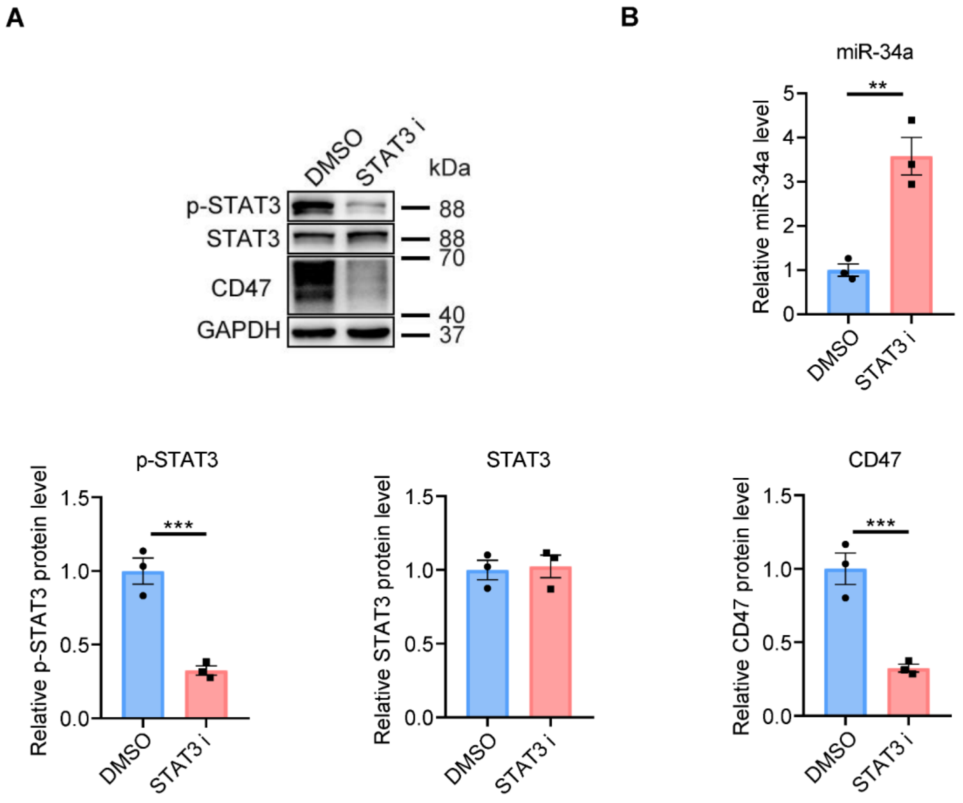

Supplementary Figure 12. Effect of the STAT3 inhibitor stattic on CD47 and miR-34a expression in lung cancer cells. (**A**) Immunoblotting analysis of the expression levels of p-STAT3, total STAT3 and CD47 in H358 cells treated with DMSO or the STAT3 inhibitor stattic. Top panel: representative blots. Bottom panel: quantification results (n = 3 per group). (**B**) Quantitative RT-PCR analysis of the relative miR-34a levels in H358 cells treated with DMSO or the STAT3 inhibitor (n = 3 per group). Data are shown as the mean ± SEM. **P < 0.01 and ***P < 0.001, by unpaired t test (**B**).

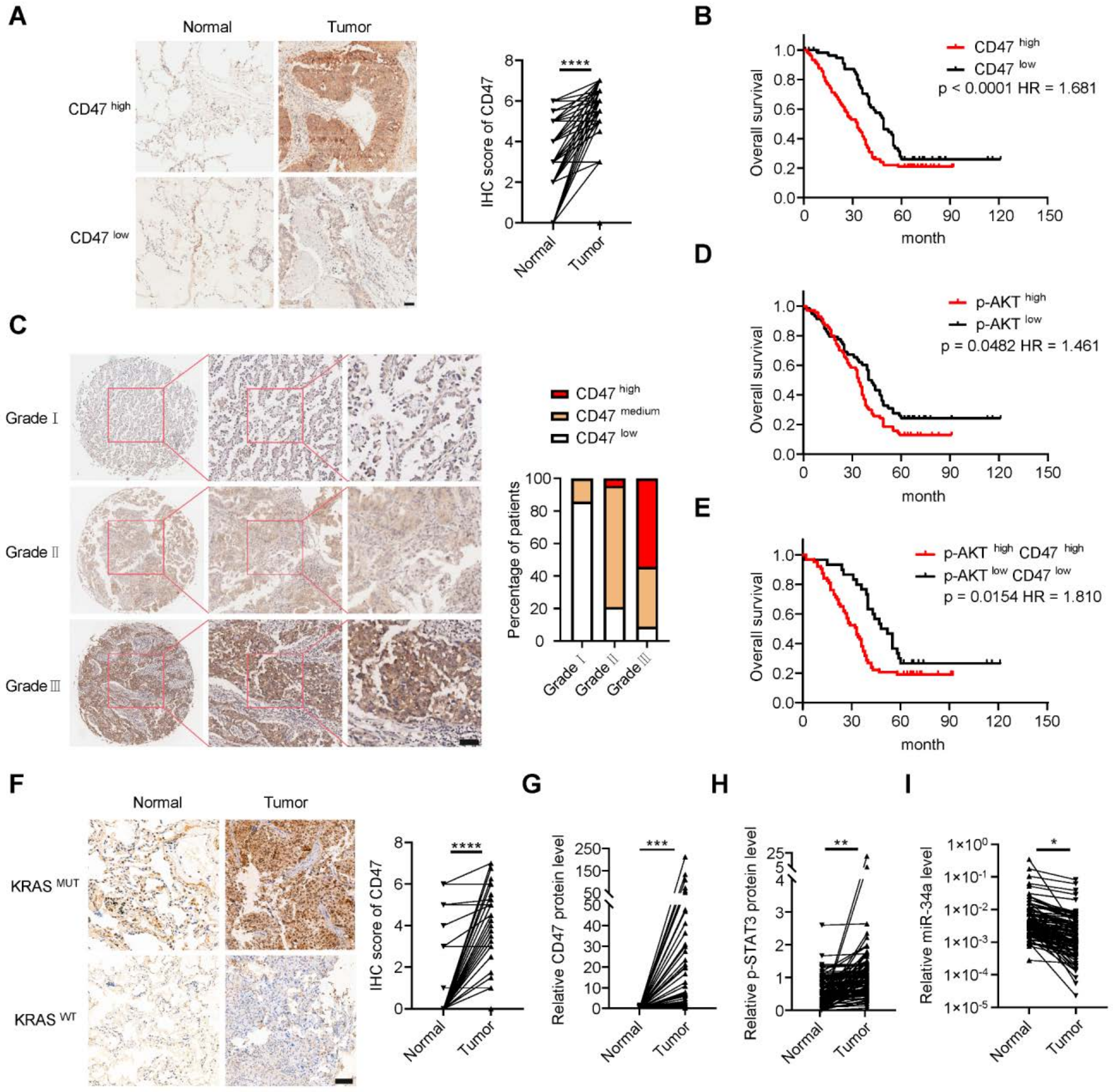

Supplementary Figure 13. Characterization of the expression patterns of CD47, p-STAT3 and miR-34a in lung adenocarcinoma patients. (**A-E**) IHC analysis of CD47 protein in the first patient cohort containing 157 lung adenocarcinoma samples. (**A**) IHC staining of CD47 protein in paired lung adenocarcinoma and normal adjacent tissue samples. Left panel: representative IHC images. Scale bar, 20 μm. Right panel: IHC scores (n = 157). (**B**) Kaplan–Meier curves were generated to analyze the association of CD47 protein expression with the overall survival of the lung adenocarcinoma patients. Patients were stratified into high or low CD47 expression groups by the median value (n = 99 and 58, respectively). (**C**) IHC staining of CD47 protein in the lung adenocarcinoma samples with different grades. Left panel: representative IHC images. Scale bar, 20 μm. Right panel: bar graph showing the percentages of patients in the grade I, II and III groups (n = 7, 93 and 57, respectively). The CD47 expression levels were stratified based on IHC scores (low: 1–4; medium: 5–6; high: 7–10). (**D**) Kaplan–Meier curves were generated to analyze the association of p-AKT protein expression with the overall survival of the lung adenocarcinoma patients. The patients were stratified into high or low p-AKT expression groups by the median value (n = 59 and 98, respectively). (**E**) Kaplan–Meier analysis of the

association between p-AKT and CD47 co-expression and overall survival of lung adenocarcinoma patients. Patients were grouped according to a positive CD47 and p-AKT co-expression pattern (both > median value) or negative (both < median value) co-expression pattern (n = 51 and 30, respectively). (**F**) IHC staining of CD47 protein in paired lung adenocarcinoma and normal adjacent tissue samples derived from the second patient cohort containing 40 lung adenocarcinoma samples (28 $KRAS^{WT}$ and 12 $KRAS^{MUT}$). Left panel: representative IHC images. Scale bar, 20 μm. Right panel: IHC scores (n = 40). (**G-H**) Western blot analyses of the expression levels of CD47 and p-STAT3 proteins in the paired lung adenocarcinoma and normal adjacent tissue samples derived from the third patient cohort containing 100 lung adenocarcinoma samples (70 $KRAS^{WT}$ and 30 $KRAS^{MUT}$). The quantification results are shown (n = 100 per group). (**I**) Quantitative RT-PCR analysis of the miR-34a levels in the paired lung adenocarcinoma and normal adjacent tissue samples derived from the third patient cohort (n = 100 per group). Data are shown as the mean ± SEM. ***P < 0.001 and ****P < 0.0001, by paired t test (**A, F-I**), survival analysis were conducted with log-rank (Mantel-Cox) test (**B, D-E**).

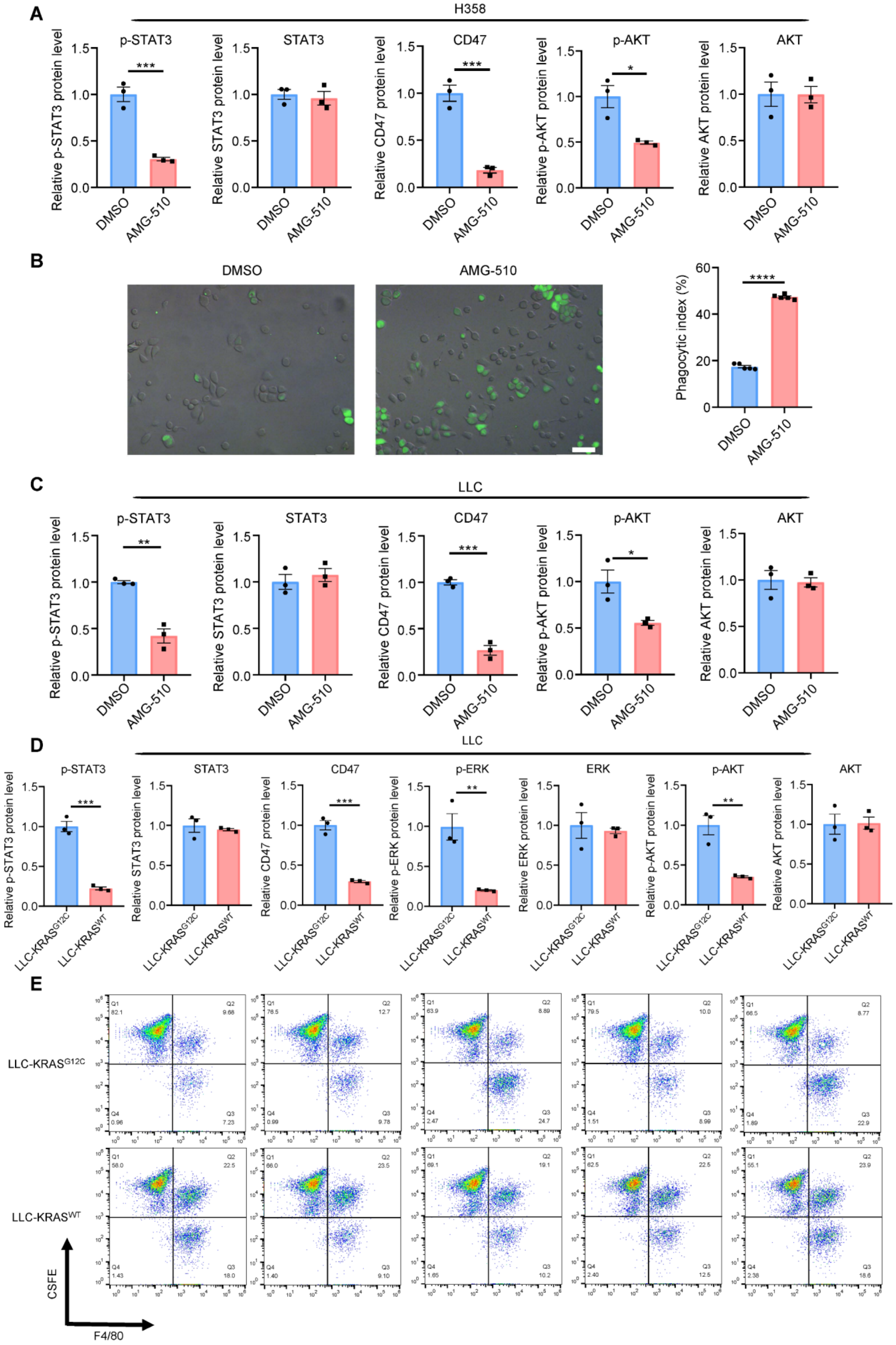

A
H358
p-STAT3
STAT3
CD47
p-AKT
AKT
Relative p-STAT3 protein level
Relative STAT3 protein level
Relative CD47 protein level
Relative p-AKT protein level
Relative AKT protein level
DMSO
AMG-510
B
DMSO
AMG-510
Phagocytic index (%)
C
LLC
D
LLC
p-ERK
ERK
Relative p-ERK protein level
Relative ERK protein level
LLC-KRAS^{G12C}
LLC-KRAS^{WT}
E
LLC-KRAS^{G12C}
LLC-KRAS^{WT}
CSFE
F4/80

Supplementary Figure 14. The KRAS^{G12C} inhibitor AMG 510 renders H358 and LLC cells sensitive to phagocytosis by macrophages. (**A**) Effect of the KRAS^{G12C} inhibitor AMG 510 on the expression levels of p-STAT3, total STAT3 and CD47 in H358 cells. Cells were treated with AMG 510 for 24 h and subjected to immunoblotting. Quantification results (n = 3 per group) are shown. (**B**) Fluorescence microscopy of the macrophage phagocytosis of the eGFP-labeled H358 cells treated with AMG 510 for 24 h before coculture with human peripheral blood monocyte-derived macrophages. Left panel: representative fluorescence images. Scale bar, 50 μm. Right panel: phagocytic index (n = 5 per group). (**C**) Effect of the KRAS^{G12C} inhibitor AMG 510 on the expression levels of p-STAT3, total STAT3 and CD47 in the LLC cells. Cells were treated with AMG 510 for 24 h and subjected to immunoblotting. Quantification results (n = 3 per group) are shown. (**D**) The expression levels of p-STAT3, total STAT3, CD47 and KRAS downstream effectors in LLC cells with heterozygous *Kras*^{G12C} or homozygous *Kras*^{WT} that were established by CRISPR/Cas9 mediated gene knock-in. (**E**) Effect of KRAS manipulation on macrophage phagocytosis of *Kras*^{G12C} or *Kras*^{WT} LLC cells. LLC cells were labeled with CFSE and subjected to the in vitro phagocytosis assay. Representative FACS result for each group is shown. Data are shown as the mean ± SEM. *P < 0.05, **P < 0.01, ***P < 0.001 and ****P < 0.0001, by unpaired t test (**A-D**).

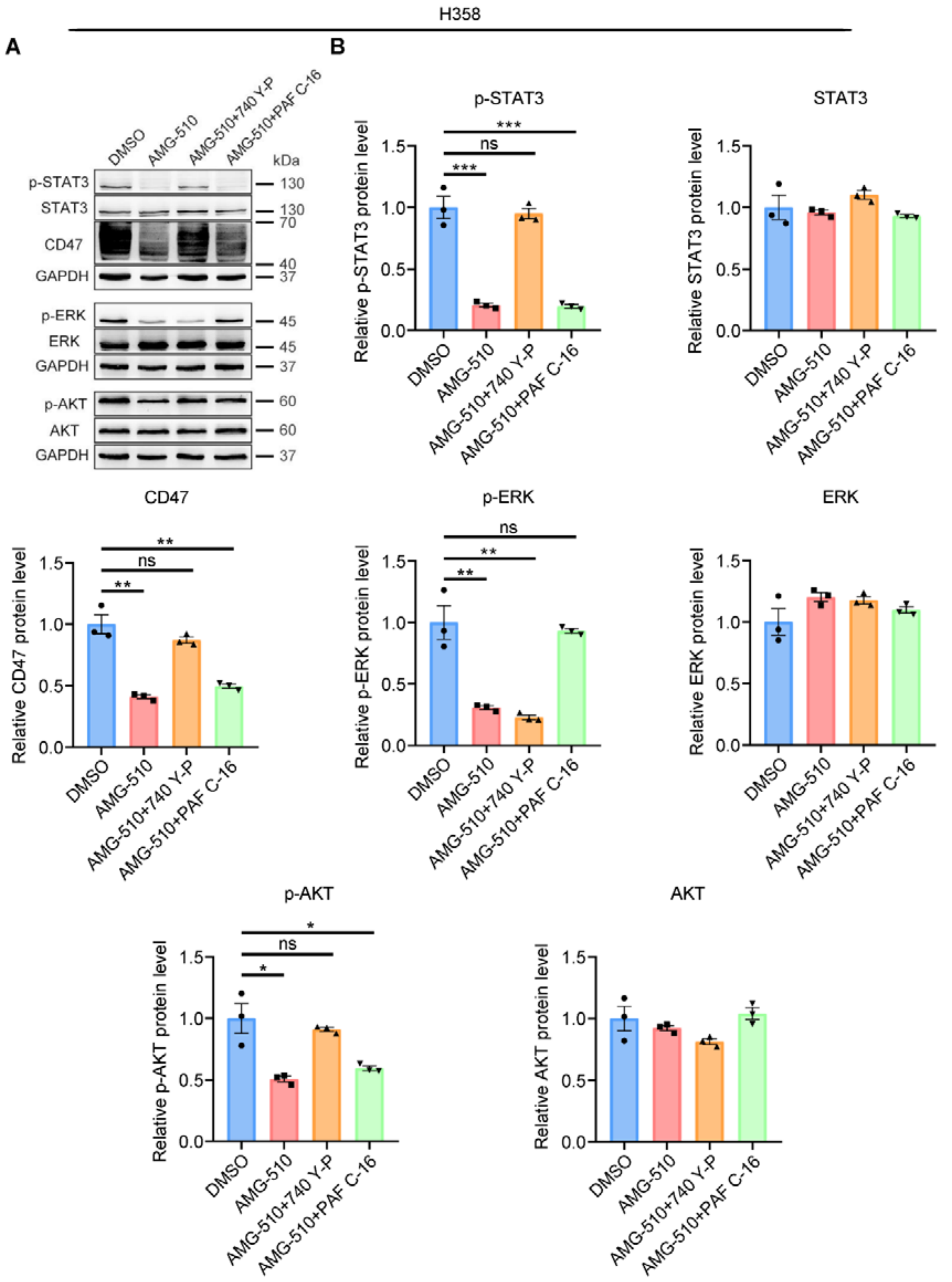

Supplementary Figure 15. *KRAS* mutations upregulate CD47 expression via the PI3K-STAT3 pathway. (**A**) Western blot analysis of the expression levels of p-STAT3, total STAT3, CD47 and KRAS downstream effectors in H358 cells treated with DMSO, AMG 510 alone, AMG 510 plus PI3K agonist or AMG 510 plus MAPK agonist. (**B**) quantification results of (**A**) (n = 3 per group) are shown. Data are shown as the mean ± SEM. *P < 0.05, **P < 0.01, ***P < 0.001 by one-way ANOVA (**B**).

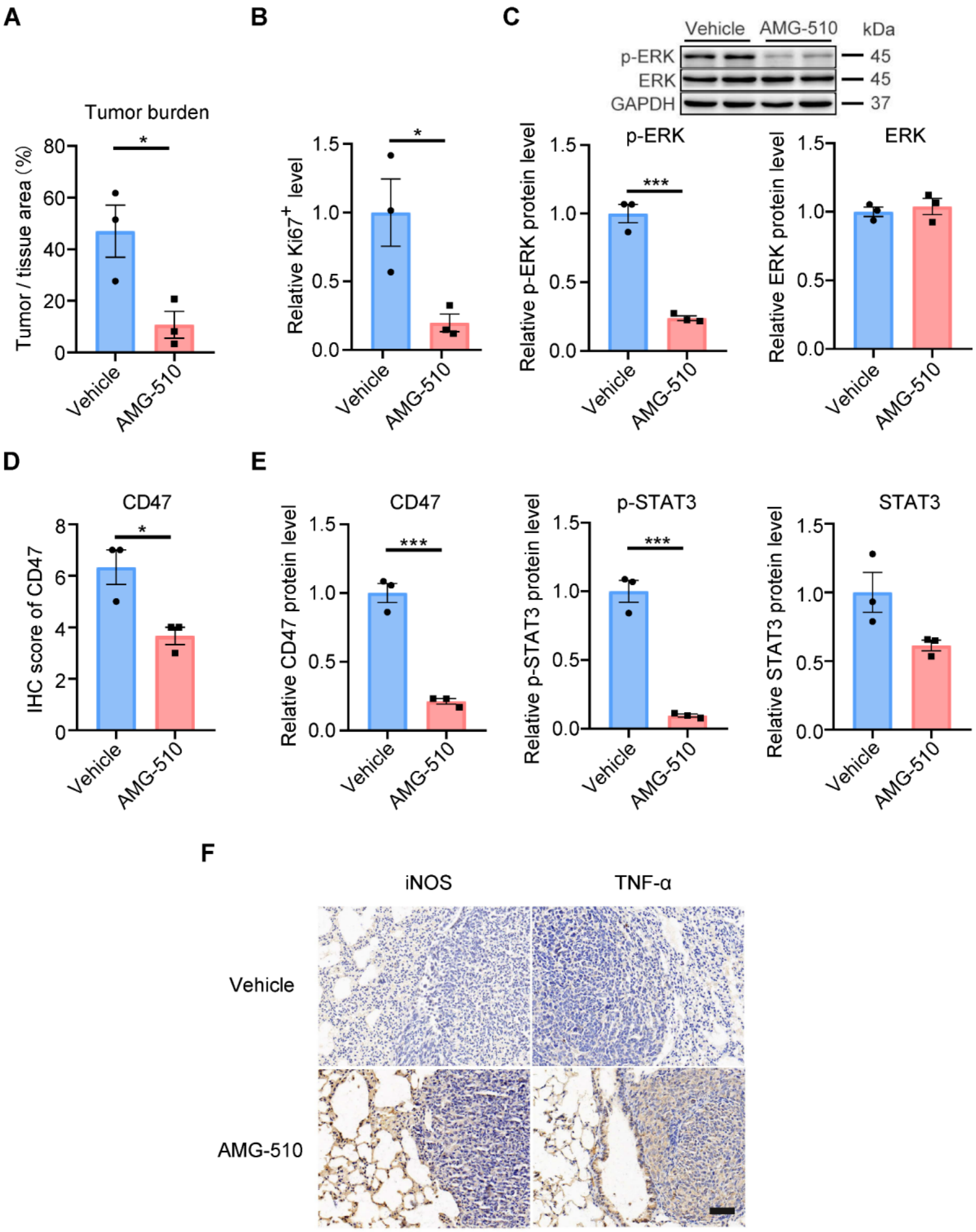

Supplementary Figure 16. The KRAS^{G12C} inhibitor AMG 510 restores innate immune surveillance in vivo. C57BL/6 immunocompetent mice were tail vein injected with LLC (*Kras^{G12C}*) mouse lung cancer cells to establish a lung colonization model. After tumor formation, the mice were administered vehicle control or AMG 510 via oral gavage for 8 days. The mice were then monitored to evaluate tumor growth, CD47 expression and macrophage phagocytosis and infiltration. (**A**) Quantification of tumor burden from H&E staining of the lung tumor sections (n = 3 per group). (**B**) Quantification of Ki67 levels from IHC staining of the lung tumor sections (n = 3 per group). (**C**) Western blot analysis of the expression levels of p-ERK and total ERK in the xenograft tumors proving KRAS target engagement. Top panel: representative blots. Bottom panel:

quantification results (n = 3 per group). (**D**) IHC staining of CD47 in the xenograft tumor sections. Quantitative analysis of the IHC scores is shown (n = 3 per group). (**E**) Western blot analysis of the expression levels of CD47, p-STAT3 and total STAT3 in the xenograft tumors. Quantification results (n = 3 per group) are shown. (**F**) Representative IHC staining of the M1 macrophage markers iNOS and TNF-α in the tumor sections. Scale bar, 20 μm. Data are shown as the mean ± SEM. *P < 0.05 and ***P < 0.001, by unpaired t test (**A-E**).

Supplementary Table 1. Significantly changed miRNAs between lung tumors and normal lung tissues of *Kras*$^{LSL\text{-}G12D/+}$ mice.

miRNAs	Mean reads in normal tissue	Mean reads in tumor	log_2 (fold-change)	p-value
mmu-miR-34a-5p	3008.628	761.357	-1.982	1.68×10^{-5}
mmu-miR-128-3p	1682.139	690.994	-1.283	8.01×10^{-3}
mmu-miR-146b-5p	113045.520	50492.338	-1.163	5.78×10^{-4}
mmu-miR-181d-5p	3088.356	1107.254	-1.479	2.74×10^{-5}
mmu-miR-340-3p	1421.727	386.605	-1.877	1.90×10^{-7}
mmu-miR-423-3p	2605.644	1078.037	-1.273	8.30×10^{-3}
mmu-miR-674-3p	1658.224	560.421	-1.565	7.45×10^{-3}
mmu-miR-1843b-3p	891.007	321.145	-1.472	6.44×10^{-3}
mmu-miR-122-5p	484.023	2833.446	2.551	9.69×10^{-5}
mmu-miR-151-5p	4295.203	12893.662	1.586	1.22×10^{-5}
mmu-miR-214-3p	2362.681	5859.413	1.310	1.99×10^{-5}
mmu-miR-221-3p	2716.599	7065.536	1.379	8.52×10^{-7}

Stringent threshold and significance criterion: mean reads > 500, log_2 (fold-change) > 1 and $P < 0.01$.

Supplementary Table 2. Demographic and clinical information of the lung adenocarcinoma samples.

A. Mutation status of 18 lung adenocarcinoma samples (12 *KRAS*^{WT} and 6 *KRAS*^{MUT}).

Patient tissue samples	*KRAS* mutation	Other mutations
Lung adenocarcinoma #1	WT	*EGFR-19del*
Lung adenocarcinoma #2		*EGFR-19del, TP53*
Lung adenocarcinoma #3		*EGFR-L858R, CDKN2A, TP53, MYCamp、EGFRamp, SESN2*
Lung adenocarcinoma #4		*TP53, CALR*
Lung adenocarcinoma #5		*ALK-EML4fusion, HIST1H1C, RAD54L*
Lung adenocarcinoma #6		*EGFR-19del, MDM2amp*
Lung adenocarcinoma #7		*EGFR-L858R, NT5C2*
Lung adenocarcinoma #8		*EGFR-19del, ABCB1, CTNNB1, LTK, MAGI2, PMS2, ETV1, EPHB1, CDH2*
Lung adenocarcinoma #9		*EGFR-19del*
Lung adenocarcinoma #10		*MAP2K1, TSC1, PDGFRA, TYRO3*
Lung adenocarcinoma #11		*EGFR-G719A/I706T, SOX17, FLT1, INSR*
Lung adenocarcinoma #12		*MDM2amp, MYCamp, GABRA6*
Lung adenocarcinoma #13	MUT	*KRAS*^{G12V}
Lung adenocarcinoma #14		*KRAS*^{G12A}
Lung adenocarcinoma #15		*KRAS*^{G12C}*, PIK3CA, SMAD4*
Lung adenocarcinoma #16		*KRAS*^{G13D}
Lung adenocarcinoma #17		*KRAS*^{G12C}*, HER2, PIK3CA*
Lung adenocarcinoma #18		*KRAS*^{G12D}*, TP53*

B. Detailed information of lung adenocarcinoma samples in the first cohort (commercial tissue microarray).

Characteristic		Cases
Gender	Male	82
	Female	75
Age	≥50	145
	< 50	12
Pathological Classification	Lung adenocarcinoma	157
TNM	I	55
	II, III	102
Pathology Grade	I	9
	II, III	148

C. Detailed information of lung adenocarcinoma samples in the second cohort (homemade tissue microarray).

Characteristic		Cases
Gender	Male	26
	Female	14
Age	≥50	35
	< 50	5
Pathological Classification	Lung adenocarcinoma	40
TNM	I	10
	II, III	30
Pathology Grade	I	16
	II, III	24
KRAS type	Mut	12
	WT	28

D. Detailed information of lung adenocarcinoma samples in the third cohort (100 individual lung adenocarcinoma samples).

Characteristic		Cases
Gender	Male	47
	Female	53
Age	≥50	87
	< 50	13
Pathological Classification	Lung adenocarcinoma	100
TNM	I	56
	II, III	44
Pathology Grade	I	52
	II, III	48
KRAS type	Mut	30
	WT	70

Supplementary Table 3. Antibody list.

Antibodies	Specificities	Provider	Cat#	Dilution ratio
F4/80	Human, Mouse	eBioscience, CA, USA	17-4801-80	2 μg/test
KRAS	Human, Mouse	Abcam, Cambridge, UK	ab172949	1:1000
Ras（G12D）	Human, Mouse	Cell Signaling Technology, MA, USA	#14429	1:1000
CD47	Mouse	R&D Systems, Minneapolis, MN, USA	AF1866	1:500
CD47	Human	R&D Systems, Minneapolis, MN, USA	AF4670	1:500
p-STAT3	Human, Mouse	Cell Signaling Technology, MA, USA	# 9145S	1:1000
STAT3	Human, Mouse	Cell Signaling Technology, MA, USA	# 4904	1:1000
GAPDH	Human, Mouse	Servicebio, Wuhan, China	GB12002	1:2000
p-AKT	Human, Mouse	Cell Signaling Technology, MA, USA	# 4060S	1:1000
p-ERK	Human, Mouse	Cell Signaling Technology, MA, USA	#4370	1:1000
ERK	Human, Mouse	Cell Signaling Technology, MA, USA	#4695	1:1000
CD11b	Human, Mouse	Proteintech Group, Rosemont, IL, USA	66519-1-lg	1:200
CD206	Human, Mouse	Abcam, Cambridge, UK	ab64693	1:2000
IL-10	Human, Mouse	Proteintech Group, Rosemont, IL, USA	20850-1-AP	1:200
iNOS	Human, Mouse	Servicebio, Wuhan, China	GB11119	1:200
TNF-α	Human, Mouse	Proteintech Group, Rosemont, IL, USA	60291-1-lg	1:200

Supplementary Table 4. Sequences of synthetic siRNAs, miRNA mimics and antisense strands.

Synthetic RNAs	Sequence
KRAS siRNA-1	Forward: CGAAUAUGAUCCAACAAUAdTdT<br>Reverse: UAUUGUUGGAUCAUAUUCGdTdT
KRAS siRNA-2	Forward: CCGACAAUACAGAUUGAAAdTdT<br>Reverse: UUUCAAUCUGUAUUGUCGGdTdT
KRAS siRNA-3	Forward: GGACUUAGCAAGAAGUUAUdTdT<br>Reverse: AUAACUUCUUGCUAAGUCCdTdT
miR-34a-5p mimic	Forward: UGGCAGUGUCUUAGCUGGUUGU<br>Reverse: ACAACCAGCUAAGACACUGCCA
miR-34a-5p antisense	ACAACCAGCUAAGACACUGCCA

Supplementary Table 5. Primer list.

Genes	Species	Real-time PCR primers
KRAS	Human	Forward: CCCAGGTGCGGGAGAGA<br>Reverse: CCCTCCCCAGTCCTCATGTA
CD47	Human	Forward: AGAAGGTGAAACGATCATCGAGC<br>Reverse: CTCATCCATACCACCGGATCT
CD47	Mouse	Forward: TGGTGGGAAACTACACTTGCG<br>Reverse: CGTGCGGTTTTTCAGCTCTAT
GAPDH	Human	Forward: TGTGGGCATCAATGGATTTGG<br>Reverse: ACACCATGTATTCCGGGTCAAT
GAPDH	Mouse	Forward: TGGATTTGGACGCATTGGTC<br>Reverse: TTTGCACTGGTACGTGTTGAT

miRNAs	Stem-loop RT primers	Real-time PCR primers
U6	GTCGTATCCAGTGCAGGGTCCGAGGTA<br>TTCGCACTGGATACGACAAAATA	Forward: CAAATTCGTGAAGCGTTCCA<br>Reverse: AGTGCAGGGTCCGAGGTATT
mmu-miR-34a-5p	GTCGTATCCAGTGCAGGGTCCGAGGTA<br>TTCGCACTGGATACGACACAACC	Forward: CGCGTGGCAGTGTCTTAGCT<br>Reverse: AGTGCAGGGTCCGAGGTATT
mmu-miR-181d-5p	GTCGTATCCAGTGCAGGGTCCGAGGTA<br>TTCGCACTGGATACGACACCCAC	Forward: CGCGAACATTCATTGTTGTCG<br>Reverse: AGTGCAGGGTCCGAGGTATT
mmu-miR-146b-5p	GTCGTATCCAGTGCAGGGTCCGAGGTA<br>TTCGCACTGGATACGACAGCCTA	Forward: CGCGTGAGAACTGAATTCCA<br>Reverse: AGTGCAGGGTCCGAGGTATT
mmu-miR-122-5p	GTCGTATCCAGTGCAGGGTCCGAGGTA<br>TTCGCACTGGATACGACCAAACA	Forward: CGCGTGGAGTGTGACAATGG<br>Reverse: AGTGCAGGGTCCGAGGTATT
mmu-miR-128-3p	GTCGTATCCAGTGCAGGGTCCGAGGTA<br>TTCGCACTGGATACGACAAAGAG	Forward: CGCGTCACAGTGAACCGGT<br>Reverse: AGTGCAGGGTCCGAGGTATT
mmu-miR-340-3p	GTCGTATCCAGTGCAGGGTCCGAGGTA<br>TTCGCACTGGATACGACGCTATA	Forward: GCGCGTCCGTCTCAGTTACTT<br>Reverse: AGTGCAGGGTCCGAGGTATT
mmu-miR-151-5p	GTCGTATCCAGTGCAGGGTCCGAGGTA<br>TTCGCACTGGATACGACACTAGA	Forward: CGCGTCGAGGAGCTCACAG<br>Reverse: AGTGCAGGGTCCGAGGTATT
mmu-miR-214-3p	GTCGTATCCAGTGCAGGGTCCGAGGTA<br>TTCGCACTGGATACGACACTGCC	Forward: GCGACAGCAGGCACAGACA<br>Reverse: AGTGCAGGGTCCGAGGTATT
mmu-miR-221-3p	GTCGTATCCAGTGCAGGGTCCGAGGTA<br>TTCGCACTGGATACGACGAAACC	Forward: CGCGAGCTACATTGTCTGCTG<br>Reverse: AGTGCAGGGTCCGAGGTATT
